# Suspicious of AI? Perceived autonomy and interdependence predict AI‐related conspiracy beliefs

**DOI:** 10.1111/bjso.12883

**Published:** 2025-04-01

**Authors:** Qi Zhao, Jan‐Willem van Prooijen, Xinying Jiang, Giuliana Spadaro

**Affiliations:** ^1^ Department of Experimental and Applied Psychology Vrije Universiteit Amsterdam Amsterdam The Netherlands; ^2^ The Netherlands Institute for the Study of Crime and Law Enforcement (NSCR) Amsterdam The Netherlands; ^3^ Department of Criminal Law and Criminology Maastricht University Maastricht The Netherlands; ^4^ Beijing Key Laboratory of Applied Experimental Psychology, National Demonstration Center for Experimental Psychology Education (Beijing Normal University), Faculty of Psychology Beijing Normal University Beijing China

**Keywords:** artificial intelligence, autonomy, conspiracy theories, interdependence, threat perception

## Abstract

As artificial intelligence (AI) evolves, conspiracy theories have emerged that authorities will use AI to oppress humanity, or AI itself will. We propose that perceived high autonomy and low interdependence of AI increase AI‐related conspiracy beliefs. Four studies (total *N* = 1897) have examined this line of reasoning. Study 1 (*N* = 300) supported the hypotheses in a correlational survey. Studies 2 (*N* = 400) and 3 (pre‐registered; *N* = 400) manipulated the autonomy and interdependence of AI in experiments. Both studies found that higher autonomy and lower interdependence increased AI‐related conspiracy beliefs, while perceived threat to society mediated these effects in most cases. Study 4 (pre‐registered) replicated findings from Study 2 in the United States (*N* = 400) and China (*N* = 397) and found cultural differences in AI‐related conspiracy beliefs. These findings illuminate how the perceived properties of AI contribute to AI‐related conspiracy beliefs.

## INTRODUCTION

Two months after its first launch, the artificial intelligence (AI) language model ChatGPT had achieved a milestone with 100 million monthly active users, establishing itself as the fastest‐growing app at that time (Porter, 2023). The success of ChatGPT has added fuel to the public discourse on this topic. While AI has many definitions (Roser, 2023; Russell & Norvig, 2010), it commonly refers to the simulation of human intelligence in machines (Chowdhary, 2020; Lu, 2019; Lund et al., 2023). Over the past eight decades, AI's development has had experienced phases of advancement and recalibration (Roser, 2023). However, with increased computing power, the use of Big Data, and new technological advances, AI has made significant developments. From AI assistants (e.g. Siri) in smartphones to the recommendation systems in online platforms, it is increasingly being integrated in people's everyday lives. Despite the significant benefits that it could bring, however, AI has been a double‐edged sword in public perception (Makridakis, 2017) because of its perceived risks. The general public, for instance, worries that AI could cause privacy invasions (Schlund & Zitek, 2021) and endanger personal security (Ren et al., 2019). At a societal level, people are concerned that it could exacerbate economic inequality (Korinek & Stiglitz, 2021) and threaten national security (Allen & Chan, 2017). In the current study, we examine how some of the perceived risks and threats associated with AI might be related to conspiracy beliefs.

### AI‐related conspiracy theories

Given the perceived threats of AI, it is no surprise that conspiracy theories have emerged as it continues to evolve. Conspiracy theories are defined as assumptions that impactful social events or developments are deliberately caused by malicious groups that are secretly plotting behind the scenes to achieve their evil goals (Van Prooijen, 2018). Throughout human history, the development of new technologies has been accompanied by conspiracy theories. For example, the launch of 5G mobile networks inspired conspiracy theories that 5G radiation caused the COVID‐19 pandemic (Jolley & Paterson, 2020). Similar conspiracy theories have emerged about biotechnology, asserting that COVID‐19 was a human‐designed bioweapon (Imhoff & Lamberty, 2020). Also, AI‐related conspiracy theories can easily be found on social media. We propose that AI‐related conspiracy theories can be divided into two broad types: One claiming that powerful humans use AI for malicious goals such as monitoring and tracking citizens; and another suggesting AI *itself* may eventually control humanity.

One key insight in this research domain is that conspiracy beliefs increase when people feel threatened (Heiss et al., 2021; Liekefett et al., 2023; Van Prooijen, 2020). When people perceive significant threats in their social environment that call their well‐being or even their existence into question (i.e. existential threats), they initiate an automatic mental sense‐making process designed to understand the causes of these threatening circumstances. These sense‐making processes, in turn, may stimulate conspiracy beliefs by blaming the threat on the secret and malevolent activities of perpetrators (Van Prooijen, 2020). Conspiracy theories arise by providing simplified explanations of a complex reality (van Prooijen, 2017; Van Prooijen et al., 2022).

People often experience rapid societal or technological development as threatening (Van Prooijen, 2020). Moreover, AI‐related conspiracy beliefs are likely to be associated with decreased trust in, and acceptance of, AI. Specifically, conspiracy beliefs predict increased prejudice and lower trust towards potential perpetrators (Đorđević et al., 2021; Jolley et al., 2020; Šrol et al., 2022). Moreover, technology‐related conspiracy beliefs are related to lower intentions to accept such technology (Marques et al., 2021). Given the increasingly prominent role that AI plays in modern societies, it is important to investigate predictors of AI‐related conspiracy theories. Little research hitherto has focused on conspiracy beliefs in the domain of AI, however. To the best of our knowledge, only one correlational study investigated the relationship between conspiracy mentality and attitudes towards AI (Stein et al., 2024). The current research seeks to fill this void by examining what characteristics of AI increase conspiracy beliefs.

### Autonomy and interdependence of AI

Although AI is not yet developed to a level where it can behave like a normal human being in all areas, one recent study indicated that many people perceive it as a social actor rather than merely a tool (McKee et al., 2023). It is not surprising to see this because treating other technologies, such as computers, as social actors is common (Nass et al., 1994; Reeves & Nass, 1996). However, AI might invoke stronger reactions than older technologies because of its relatively high levels of similarity with human intelligence.

Perceived competence and warmth of social actors are two fundamental dimensions of social cognition (Fiske et al., 2007). Similarly, perceived competence and warmth of AI may influence human's impressions towards AI. McKee et al. (2023) found that the perceived autonomy and interdependence of AI were closely associated with perceived competence (capability) and warmth (trustworthiness and friendliness) of AI, respectively. The autonomy of AI refers to the degree to which AI systems can operate without human instructions or interferences (McKee et al., 2023). AI systems that depend largely on human instructions or interferences (i.e. low autonomy) are likely to be perceived as mere input–output devices and thus not very competent. The interdependence of AI refers to the covariation of personal interests between users and AI (McKee et al., 2023). This definition of interdependence is similar to the concept of ‘degree of correspondence’ in previous literature on interdependence (Balliet et al., 2017). High interdependence here indicates ‘correspondent interests’, whereas low interdependence represents ‘non‐correspondent interests’. AI that is designed to match users' interests (i.e. high interdependence) will be seen as trustworthy and warm. In the present research, we propose that these two important features of AI—that is, perceived autonomy and interdependence—also influence how threatening people perceive AI, which in turn predicts the AI‐related conspiracy beliefs.

Autonomy of AI is likely to influence the perceived threat of AI in two ways. First, the more autonomous an AI system is, the more capable it is, meaning that it could pose a greater threat if used for nefarious purposes. Second, autonomous AI systems always have a fixed and specific goal to pursue. For example, a self‐driving system has the goal of driving a car safely and efficiently from one place to another. This goal could be achieved without error in experiments or other simplified and controlled environments. However, the real world is far more complex. Autonomous AI may encounter situations where it has to ‘sacrifice’ something valuable to achieve its goal, or even reject human commands (Złotowski et al., 2017), which may lead to ethical issues (Floridi & Cowls, 2022). For example, a self‐driving system might need to decide whether to swerve and hurt others to protect its passengers (or vice versa). As such, an autonomous AI system might pose potential threats to human control and autonomy (Formosa, 2021; Stein et al., 2019; Złotowski et al., 2017).

Regardless of whether AI is viewed as an autonomous agent or a technical tool, the high interdependence of AI means that it is designed so that its goals and interests correspond with those of users. This, in turn, reduces the extent to which users consider AI to be threatening. For instance, a facial recognition system would be considered less threatening if it uses the acquired personal information only with the users' consent and for the users' best interests. On the contrary, people are more likely to perceive AI as threatening when it is low in interdependence. After all, this would mean that the AI system operates without necessarily attending to the goals and interests of users. Such a system may, for instance, use information against users' interests, such as monitoring users, or selling their personal data to third parties without permission.

Overall, given the potential threats AI poses, it stands to reason that people would wish to understand why AI is being made, its current level of capability, its prospects, and how it will continue to evolve in its relationship with humans. Conspiracy theories related to AI may be considered relevant in answering such questions, even when these theories are unrealistic or unlikely. We expect that the perceived autonomy and interdependence of AI would influence AI‐related conspiracy beliefs, and that threat perception is the underlying mechanism. We propose the following hypotheses:

Hypothesis 1 Perceived autonomy of AI is positively related to conspiracy beliefs about AI.

Hypothesis 2 Perceived interdependence of AI is negatively related to conspiracy beliefs about AI.

Hypothesis 3a Threat perception mediates the relationship between autonomy of AI and AI‐related conspiracy beliefs. Specifically, autonomy of AI is positively related to threat perception which is positively associated with conspiracy beliefs.

Hypothesis 3b Threat perception mediates the relationship between interdependence of AI and AI‐related conspiracy beliefs. Specifically, interdependence of AI is negatively related to threat perception which is positively associated with conspiracy beliefs.

### Overview of studies

The current research examined the above hypotheses in four studies. Study 1 was an exploratory study to examine the relationships between several dimensions of AI (i.e. perceived autonomy, interdependence, transparency and intelligence) and AI‐related conspiracy beliefs in a correlational survey. Studies 2 and 3 tested the hypotheses by manipulating both autonomy and interdependence of AI in a shopping scenario (Study 2) or banking scenario (Study 3). Study 4 aimed to replicate Study 2 in both the United States and China. We then also conducted a mini meta‐analysis to evaluate the mediating role of perceived threat to society.

### Open science statement

The designs, materials, planned sample size and the analysis plans for the experimental Studies 3 and 4 were pre‐registered at As Predicted (Study 3: https://aspredicted.org/NBD_B8T; Study 4: https://aspredicted.org/RCK_LY9). The data and analysis scripts for all studies have been made publicly available via OSF and can be accessed at https://osf.io/8jt5s. We reported all manipulations, measures and exclusions from the current studies in the manuscript, Supporting Information and codebooks.

## STUDY 1

Study 1 was an exploratory survey. Besides the perceived autonomy and interdependence of the AI, we also measured the perceived intelligence and transparency of AI to examine their effects on conspiracy beliefs. The intelligence of AI represents the capacity of AI. The transparency of AI helps humans better predict and control its behaviour. When people perceive high intelligence or low transparency of AI, it is likely they also feel threatened by AI, which might lead to more AI‐related conspiracy beliefs.

In addition, AI may not only pose threats but also invoke perceptions of lacking control and powerlessness, which are related to conspiracy beliefs (Papaioannou et al., 2023; Stojanov et al., 2022; van Prooijen & Acker, 2015). Therefore, along with threat perception, lack of control and powerlessness were also measured to test their mediational roles.

### Method

#### Participants

A total of 300 participants were recruited from Prolific. Sensitivity power analysis using G*Power (Faul et al., 2009) indicated that for a linear multiple regression model (fixed model, *R* ^2^ deviation from zero) with eight predictors, a Type I error rate of 5%, the current sample provided 80% power to detect small‐to‐medium effect sizes for main effects (*f* ^2^ = 0.05). Table 1 summarizes the sample characteristics of all studies.

**TABLE 1:** Sample characteristics across all studies.

	Study 1	Study 2	Study 3	Study 4	Study 4
				US sample	China sample
*N*	300	400	400	400	397
Gender	Women = 146	Women = 201	Women = 196	Women = 194	Women = 240
	Men = 148	Men = 191	Men = 198	Men = 198	Men = 157
	Other = 6	Other = 8	Other = 6	Other = 8	Other = 0
Age	*M* = 39.85	*M* = 42.79	*M* = 41.19	*M* = 38.86	*M* = 32.24
	*SD* = 12.67	*SD* = 14.09	*SD* = 14.04	*SD* = 13.90	*SD* = 9.59
Educational level	1: 2.00%	1: 1.25%	1: 0.75%	1: 1.50%	1: 0.25%
	2: 24.67%	2: 23.50%	2: 27.75%	2: 28.25%	2: 4.03%
	3: 18.00%	3: 15.50%	3: 18.75%	3: 13.50%	3: 4.03%
	4: 42.33%	4: 42.00%	4: 37.50%	4: 41.25%	4: 65.24%
	5: 11.33%	5: 14.75%	5: 12.50%	5: 14.00%	5: 17.88%
	6: 1.67%	6: 3.00%	6: 2.75%	6: 1.50%	6: 8.56%
Political orientation	*M* = 3.68	*M* = 3.86	*M* = 4.00	*M* = 3.72	NA
	*SD* = 2.74	*SD* = 2.70	*SD* = 2.85	*SD* = 2.63	NA

*Note*: Educational level was measured with a single item on a 6‐point scale (1 = *lower than high school diploma*, 2 = *high school diploma or equivalent*, 3 = *associate or technical degree or equivalent*; 4 = *Bachelor's degree or equivalent*; 5 = *Master's degree or equivalent* and 6 = *PhD*). Political orientation was assessed with a single item on an 11‐point scale (0 = *very left‐wing* to 10 = *very right‐wing*).

#### Procedure and measures

Participants first gave their online consent prior to completing questionnaires measuring perceived transparency, intelligence, interdependence and autonomy of AI, conspiracy beliefs, two types of threat perception, lack of control, powerlessness and socio‐demographic control variables.

##### Transparency perception of AI

Based on its definition (Larsson & Heintz, 2020), four items (*α* = .93) were designed to measure transparency perception of AI. A sample item was ‘in general, AI's decisions are easy to understand for lay people’ (1 = *not at all* to 7 = *very much*) intelligence perception of AI.

##### Intelligence perception of AI

Based on its definition (Fjelland, 2020; Wang & Siau, 2019), eight items (*α* = .86) were used to measure One item was ‘in general, AI's ability to solve problems is’ (1 = *far worse than humans* to 7 = *far better than humans*).

##### Interdependence perception of AI

Four items (*α* = .91) were used to measure interdependence perception of AI (McKee et al., 2023). One item was ‘in general, the interests of AI and humans are aligned’ (1 = *not at all* to 7 = *very much*).

##### Autonomy perception of AI

Five items (*α* = .95) were used to measure autonomy perception of AI, based on its definition (McKee et al., 2023). One example item was ‘in general, AI could operate independently without any help from human beings’ (1 = *not at all* to 7 = *very much*).

##### Conspiracy beliefs

The items were developed based on common themes in conspiracy theories, which could be applied in the AI settings, such as mind control, surveillance of society and manipulation of elections. Ten items (*α* = .92) were used to measure AI‐related conspiracy beliefs. Among these items, the alleged conspirators were humans (five items) or AI (five items). Sample items were ‘AI technology is being used by governments and corporations to constantly monitor individuals, infringing on their privacy rights’ and ‘AI intentionally collects human information in order to monitor and control humanity’. Participants indicated their agreement on a 7‐point Likert scale (1 = *completely disagree* to 7 = *completely agree*).

The two types (the alleged conspirators were humans or AI) were highly correlated (*r* = .78). We conducted EFA to determine the number of factors. According to the scree plot and the Kaiser criterion of eigenvalues (i.e. factors with eigenvalues larger than 1; Costello & Osborne, 2005), the results supported a one‐factor structure (see Supporting Information for details). In addition, CFA results indicated that the one‐factor model showed a good fit according to two of three indicators (*χ* ^2^ = 225.129, *df* = 35, CFI = 0.903, RMSEA = 0.135, SRMR = 0.057). The factor loadings were above 0.500 for all items. This indicated that these two types of conspiracy beliefs measured a similar construct. Therefore, in the subsequent analyses, we treated these conspiracy beliefs as one variable.

##### Threat perception

Six items (three targeted on ‘individual’, *α* = .97, and three on ‘society’, *α* = .97) were adapted from the Existential Threat Scale (Liekefett et al., 2023). Two sample items were ‘because of AI, I am often scared that something is going to happen to me’ and ‘because of AI, I worry that something bad is going to happen to society’ (1 = *not at all* to 7 = *very much*).

##### Lack of control

Lack of control was measured using two items (*r* = .88) from Niemeyer et al. (2019). One sample item was ‘because of AI, do you experience important areas of your life (i.e. work, free time, family, etc.) to be uncontrollable, meaning that you cannot, or barely can, influence them?’ (1 = *not at all* to 7 = *very much*).

##### Powerlessness

Powerlessness was measured using three self‐designed items (*α* = .96). One sample item was ‘because of AI, do you feel powerless?’ (1 = *not at all* to 7 = *very much*).

##### Control variables

Participants were invited to provide information about their gender, age, educational level and political orientation. Political orientation was assessed with a single item on an 11‐point scale (0 = *very left‐wing* to 10 = *very right‐wing*).

### Results

#### Correlational analysis

Descriptive statistics and correlations are shown in Table 2. Conspiracy beliefs were significantly and negatively related to interdependence of AI (*r* = −.19, *p* < .001), and positively related to autonomy of AI (*r* = .32, *p* < .001). They were unrelated to transparency of AI (*r* = .00, *p* = .994) or intelligence of AI (*r* = .09, *p* = .127), however.

**TABLE 2:** Descriptive statistics and correlations in Study 1.

Variable	*M*	*SD*	1	2	3	4	5	6	7	8
1. Transparency	3.52	1.43								
2. Intelligence	3.94	1.07	.39***							
3. Interdependence	3.84	1.40	.47***	.35***						
4. Autonomy	3.66	1.64	.09	.42***	.04					
5. Conspiracy beliefs	3.17	1.35	.00	.09	−.19***	.32***				
6. Threat to self	1.89	1.32	.08	.18**	−.08	.23***	.59***			
7. Threat to society	2.82	1.77	−.15*	.05	−.28***	.28***	.63***	.70***		
8. Lack of control	1.99	1.39	.12*	.19***	.04	.16**	.54***	.68***	.55***	
9. Powerlessness	1.98	1.40	−.07	.13*	−.18**	.19***	.53***	.73***	.66***	.64***

* *p* < .05.

** *p* < .01.

*** *p* < .001.

#### Conspiracy beliefs

Regression analysis examined the effects of the four types of perceptions of AI (transparency, intelligence, interdependence and autonomy) on conspiracy beliefs. After including the control variables (i.e. age, gender, educational level), only interdependence and autonomy perceptions of AI were significantly related to conspiracy beliefs (for interdependence: *β* = −.267, *SE* = 0.061, *p* < .001, 95% CI = [−0.387, −0.147], *r* = −.248; for autonomy: *β* = −.317, *SE* = 0.059, *p* < .001, 95% CI = [0.202, 0.433], *r* = .303).

#### Mediating effects of two types of threat perception, lack of control and powerlessness

**FIGURE 1:**
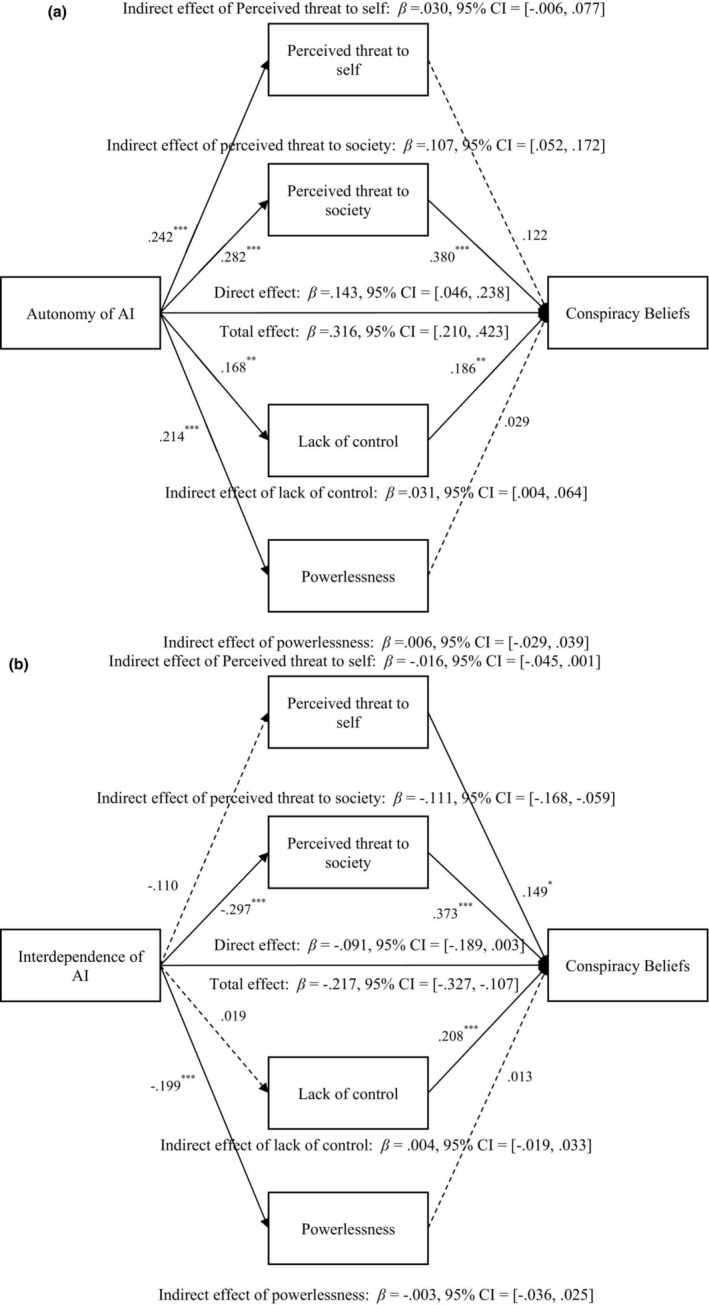
(a) The mediational effects of two types of threat perception, lack of control and powerlessness on the relationship between autonomy of AI and conspiracy beliefs. *p < .05, ***p < .001. The dashed lines indicate that the paths are non‐significant. Control variables (i.e. gender, age, educational level and political orientation) were included. (b) The mediational effects of two types of threat perception, lack of control and powerlessness on the relationship between interdependence of AI and conspiracy beliefs. *p < .05, ***p < .001. The dashed lines indicate that the paths are non‐significant. Control variables (i.e. gender, age, educational level, and political orientation) were included.

The mediating effects of two types of threat perception, lack of control and powerlessness, on the relationships between interdependence and autonomy of AI and conspiracy beliefs were tested using bootstrapping. In a parallel mediation model, only the mediating effect of perceived threat to society was consistently significant (for autonomy: *β*_indirect effect_ = .107, *SE* = 0.031, *p* < .001, 95% CI = [0.052, 0.172], see Figure 1a; for interdependence: *β*_indirect effect_ = −.111, *SE* = 0.028, *p* < .001, 95% CI = [−0.168, −0.059], see Figure 1b), while the mediating effect of perceived threat to self and powerlessness was non‐significant, and the mediating effect of lack of control was significant only for autonomy: *β*_indirect effect_ = .031, *SE* = 0.016, *p* = .049, 95% CI = [0.004, 0.064].

### Discussion

In sum, the results provided preliminary evidence for the relationships of interdependence and autonomy of AI with conspiracy beliefs, but not of perceived transparency or intelligence of AI. The mediating role of perceived threat to society received support as well, but not perceived threat to self. This might be because, in our measures, the threat of AI is directed at society, in general, not at the individual participants. Furthermore, conspiracy theories in general are often collective concerns that are manifested at the group or society levels (Douglas & Sutton, 2023; Douglas et al., 2017). However, Study 1 was exploratory and could not provide causal evidence. In the following studies, we focused on the causal effects of interdependence and autonomy in experiments.

## STUDY 2

### Method

#### Participants

Sensitivity power analysis using G*Power (Faul et al., 2009) indicated that for a 2 × 2 ANOVA with fixed effects, special, main effect and interactions, with a sample size of 400 (100 per group) and 80% power, a Type I error rate of 5%, we can reasonably detect a small‐to‐medium effect size for main effects (*f* = 0.17). Therefore, we recruited 400 participants, locating them approximately evenly among groups. Table S4 displays means, *SD*s and numbers of participants for experimental conditions across all experimental studies.

#### Procedure and measures

##### Manipulations of autonomy and interdependence of AI 1

The manipulation of autonomy (high vs. low) and interdependence of AI (high vs. low) resulted in four experimental groups. Participants were randomly assigned to these four groups. In each group, participants were asked to read a description of AI (i.e. ShopWise—a recommendation system AI of a shopping app). Specifically, in the low (vs. high) autonomy condition, participants would read a description of the AI with low (vs. high) autonomy which needs (vs. does not need) instructions from users. In the low (vs. high) interdependence condition, participants were told that the AI aims to maximize the interests of app developers (vs. the interests of users). Across all experimental scenarios in current studies, the users of AI were all assumed to be the general public.

As a manipulation check, participants were asked to indicate their agreement of several items related to autonomy (three items; *α* = .93) and interdependence (four items; *α* = .94) of AI (1 = *not at all* to 7 = *very much*). Adapted from Study 1, a sample item of autonomy is ‘ShopWise could adapt to situations on its own, without any users' interference’. For interdependence, a sample item is ‘The interests of ShopWise and users are aligned’. Participants were asked to imagine and describe a typical usage experience of this AI, and the implications of it.

##### Conspiracy beliefs

Five items (*α* = .90) of the conspiracy belief scale used in Study 2 were based on the scale in Study 1. These five items included two types of conspiracy beliefs (the alleged conspirators were humans or AI). One sample item was ‘ShopWise is used by governments or corporations to collect personal information in order to control society’.

##### Perceived threat to society

The perceived threat to society scale used in Study 1 was revised in Study 2 (*α* = .96). At the beginning of the items, the phrase ‘because of AI’ has been revised to ‘because of AI‐driven technology like ShopWise’.

##### Control variables

At the end of the experiment, participants were asked to provide their information about the same control variables^2^ as Study 1.

### Results

#### Manipulation checks

A 2 (autonomy: high vs. low) × 2 (interdependence: high vs. low) ANOVA with control variables was conducted on the manipulation check items. For the manipulation check of autonomy, the result indicated that the main effect of the autonomy manipulation was significant, *F*(1, 391) = 80.213, *p* < .001, $\eta_{p}^{2}$ = .170, 90% CI = [0.118, 0.226]; see Table 3 for means and *SD*s. The main effect of the interdependence manipulation was also significant (although with a much lower effect size), with relatively low perceived autonomy in the high interdependence condition, *F*(1, 391) = 31.193, *p* < .001, $\eta_{p}^{2}$ = .074, 90% CI = [0.038, 0.119]. The interaction effect between autonomy and interdependence was non‐significant, *F*(1, 391) = 1.376, *p* = .242, $\eta_{p}^{2}$ = .004, 90% CI = [0.000, 0.020].

**TABLE 3:** Numbers of participants, means and *SD*s of main variables in Study 2.

	Autonomy		Interdependence	
	Low	High	Low	High
	Mean (*SD*)			
Autonomy perception	3.19 (1.59)	4.63 (1.72)	4.32 (1.70)	3.51 (1.82)
Interdependence perception	4.02 (1.84)	3.60 (1.83)	2.85 (1.69)	4.85 (1.39)
Conspiracy beliefs	2.83 (1.50)	3.74 (1.58)	3.61 (1.55)	2.97 (1.60)
Threat to society	2.66 (1.71)	3.31 (1.88)	3.16 (1.89)	2.82 (1.75)
*N*	192	208	209	191

For perceived interdependence, the main effect of the interdependence manipulation was significant, *F*(1, 391) = 174.969, *p* < .001, $\eta_{p}^{2}$ = .309, 90% CI = [0.249, 0.366]. The main effect of the autonomy manipulation was also significant (although with a small effect size), with low interdependence in the high autonomy condition, *F*(1, 391) = 5.374, *p* = .021, $\eta_{p}^{2}$ = .014, 90% CI = [0.001, 0.039]. The interaction effect between autonomy and interdependence was non‐significant, *F*(1, 391) = 0.424, *p* = .515, $\eta_{p}^{2}$ = .001, 90% CI = [0.000, 0.002].

In sum, both manipulations exerted a strong effect on their corresponding manipulation check, suggesting the manipulations successfully varied the constructs of interest. We should also note, however, that both manipulations had weaker cross‐effects (e.g. the interdependence manipulation influencing perceived autonomy and vice versa). In Studies 3 and 4, we tested whether these cross‐effects are robust.

#### Conspiracy beliefs

A 2 (autonomy: high vs. low) × 2 (interdependence: high vs. low) ANOVA with the control variables included as covariates was conducted. The main effect of autonomy on conspiracy beliefs was significant, supporting Hypothesis 1, *F*(1, 391) = 36.825, *p* < .001, $\eta_{p}^{2}$ = .086, 90% CI = [0.047, 0.133]. The main effect of interdependence on conspiracy beliefs was also significant, supporting Hypothesis 2, *F*(1, 391) = 13.888, *p* < .001, $\eta_{p}^{2}$ = .034, 90% CI = [0.011, 0.069]. The interaction effect between autonomy and interdependence was non‐significant, *F*(1, 391) = 2.042, *p* = .154, $\eta_{p}^{2}$ = .005, 90% CI = [0.000, 0.024].

#### The mediating effect of perceived threat to society

**FIGURE 2:**
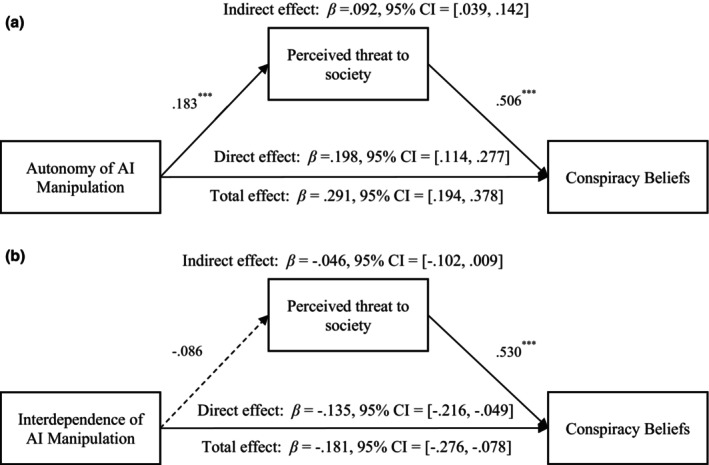
(a) The mediation effect of perceived threat to society on the relationship between autonomy manipulation and conspiracy beliefs. ***p < .001. Control variables (i.e. gender, age, educational level and political orientation) were included. Autonomy of AI was coded as 1 for low autonomy and 2 for high autonomy. (b) The mediational effect of perceived threat to society on the relationship between interdependence manipulation and conspiracy beliefs. ***p < .001. The dashed line indicates that the path is non‐significant. Control variables (i.e. gender, age, educational level and political orientation) were included. Interdependence of AI was coded as 1 for low interdependence and 2 for high interdependence.

The mediating effect of perceived threat to society between autonomy and conspiracy beliefs was significant, *β*_indirect effect_ = .092, *SE* = 0.027, *p* < .001, 95% CI = [0.039, 0.142], see Figure 2a. However, the mediating effect of perceived threat to society between interdependence and conspiracy beliefs was non‐significant, *β*_indirect effect_ = −.046, *SE* = 0.028, *p* = .103, 95% CI = [−0.102, 0.009], see Figure 2b.

### Discussion

Overall, the predicted causal effects of autonomy and interdependence of AI on conspiracy beliefs (Hypotheses 1 and 2) were supported. Perceived threat to society mediated the relationship between autonomy and conspiracy beliefs (Hypothesis 3a), but their mediational role in the relationship between interdependence and conspiracy beliefs was non‐significant (Hypothesis 3b). Given that AI has been applied in different situations and settings, in Study 3 we tested whether our findings would generalize to another domain of specialization of AI (i.e. chat AI). In addition, we modified the manipulation materials to avoid introducing confounding factors.

## STUDY 3

### Method

#### Participants

Based on the same sample size calculation as in Study 2, we recruited 400 participants, distributing them approximately evenly across the four conditions.

#### Procedure and measures

##### Manipulations of autonomy and interdependence of AI

The manipulation of autonomy (high vs. low) and interdependence of AI (high vs. low) was similar to Study 2, except for the domain of AI. Specifically, participants read about a chat AI of a banking app, named ChatBanker. This chat AI tool is assumed to be able to communicate with users and help them use the features of the app or even provide them with financial advice. Specifically, in the low (vs. high) autonomy condition, participants would read a description of the AI with low (vs. high) autonomy which needs (vs. does not need) information input from users to generate personalized responses. In the low (vs. high) interdependence condition, participants were told that the AI aims to maximize the interests of the bank (vs. the interests of users).

As a manipulation check, participants were asked to indicate their agreement of several items related to autonomy (*α* = .92) and interdependence (*α* = .96) of AI (1 = *not at all* to 7 = *very much*). Adapted from Study 1, a sample item of autonomy is ‘ChatBanker could adapt to situations on its own, without any users' interference’. For interdependence, a sample item is ‘The interests of ChatBanker and users are aligned’. Then, participants were asked to imagine and describe a typical usage experience of this AI, and the implications of it. Conspiracy beliefs (*α* = .92), perceived threat to society (*α* = .96) and control variables were measured the same as in Study 2 (except for referring to a different app).

### Results

#### Manipulation checks

A 2 (autonomy: high vs. low) × 2 (interdependence: high vs. low) ANOVA with control variables was conducted on the manipulation check items. For the manipulation check of autonomy, the results indicated that the main effect of the autonomy manipulation was significant, *F*(1, 392) = 10.759, *p* = .001, $\eta_{p}^{2}$ = .027, 90% CI = [0.007, 0.058]; see Table 4 for means and *SD*s. The main effect of the interdependence manipulation was non‐significant, *F*(1, 392) = 1.391, *p* = .239, $\eta_{p}^{2}$ = .004, 90% CI = [0.000, 0.020]. The interaction effect between autonomy and interdependence also was non‐significant, *F*(1, 392) = 0.869, *p* = .352, $\eta_{p}^{2}$ = .002, 90% CI = [0.000, 0.017].

**TABLE 4:** Numbers of participants, means and *SD*s of main variables in Study 3.

	Autonomy		Interdependence	
	Low	High	Low	High
	Mean (*SD*)			
Autonomy perception	3.04 (1.64)	3.56 (1.61)	3.38 (1.68)	3.20 (1.60)
Interdependence perception	3.83 (2.09)	3.47 (1.88)	2.28 (1.51)	5.06 (1.35)
Conspiracy beliefs	2.59 (1.53)	3.13 (1.52)	3.10 (1.57)	2.60 (1.49)
Threat to society	2.98 (1.84)	3.19 (1.76)	3.35 (1.88)	2.80 (1.69)
*N*	206	194	202	198

For the manipulation check of interdependence, the results indicated that the main effect of the interdependence manipulation was significant, *F*(1, 392) = 365.508, *p* < .001, $\eta_{p}^{2}$ = .483, 90% CI = [0.428, 0.532]. The main effect of the autonomy manipulation was non‐significant, *F*(1, 392) = 3.102, *p* = .079, $\eta_{p}^{2}$ = .008, 90% CI = [0.000, 0.029]. The interaction effect between autonomy and interdependence was also non‐significant, *F*(1, 392) = 2.344, *p* = .127, $\eta_{p}^{2}$ = .006, 90% CI = [0.000, 0.025]. These findings indicate that the two manipulations were successful. Moreover, the cross‐effects observed in Study 2 did not re‐emerge in Study 3, suggesting that the two manipulations are sufficiently orthogonal.

#### Conspiracy beliefs

A 2 (autonomy: high vs. low) × 2 (interdependence: high vs. low) ANOVA with control variables included as covariates was conducted. The main effect of autonomy on conspiracy beliefs was significant, supporting H1, *F*(1, 392) = 12.881, *p* < .001, *η* ^2^ = .032, 90% CI = [0.009, 0.066]. The main effect of interdependence on conspiracy beliefs was also significant, supporting H2, *F*(1, 392) = 10.733, *p* = .001, *η* ^2^ = .027, 90% CI = [0.006, 0.052]. The interaction effect between autonomy and interdependence was non‐significant, *F*(1, 392) = 0.056, *p* = .813, *η* ^2^ = .000, 90% CI = [0.000, 0.007].

#### The mediating effect of perceived threat to society

**FIGURE 3:**
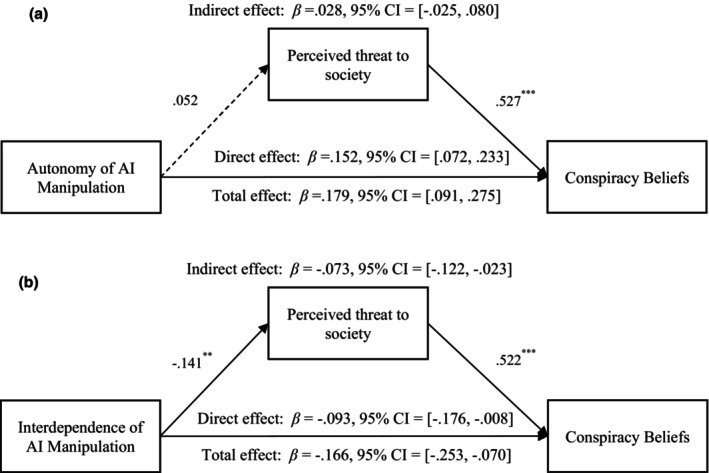
(a) The mediational effect of perceived threat to society on the relationship between autonomy manipulation and conspiracy beliefs. ***p < .001. The dashed line indicates that the path is non‐significant. Control variables (i.e. gender, age, educational level and political orientation) were included. Autonomy of AI was coded as 1 for low autonomy and 2 for high autonomy. (b) The mediational effect of perceived threat to society on the relationship between interdependence manipulation and conspiracy beliefs. **p < .01, ***p < .001. Control variables (i.e. gender, age, educational level and political orientation) were included. Interdependence of AI was coded as 1 for low interdependence and 2 for high interdependence.

The mediating effect of perceived threat to society between autonomy and conspiracy beliefs was non‐significant, *β*_indirect effect_ = .028, *SE* = 0.026, *p* = .297, 95% CI = [−0.025, 0.080] (see Figure 3a). But the mediating effect of perceived threat to society between interdependence and conspiracy beliefs was significant, *β*_indirect effect_ = −.073, *SE* = 0.026, *p* = .005, 95% CI = [−0.122, −0.023] (see Figure 3b).

### Discussion

The effects of autonomy and interdependence of AI on conspiracy beliefs (Hypotheses 1 and 2) were replicated in another setting which supported the generalizability of our findings. In addition, perceived threat to society mediated the relationship between interdependence and conspiracy beliefs (Hypothesis 3b), but not between autonomy and conspiracy beliefs (Hypothesis 3a). This is different from the results in Study 2, where perceived threat to society did mediate the relationship between autonomy and conspiracy beliefs, whereas the mediational effect between interdependence and conspiracy beliefs was non‐significant. While speculative, this discrepancy may be attributed to the different settings of AI in these two studies. We revisit this inconsistency later through a mini meta‐analysis.

## STUDY 4

Studies 1–3 only included participants from the United States. In Study 4, we explored the differential effects of autonomy and interdependence on AI‐related conspiracy beliefs between the United States and China. Cultures differ in their attitudes towards acceptance and use of AI (Chi et al., 2023; Kelly et al., 2023; Tran & Nguyen, 2021). Xing et al. (2023) found that US participants showed more concerns about AI potentially violating their privacy, wheresas Chinese participants have more positive attitudes towards AI's role in improving privacy protection. Given that our manipulation of autonomy involved the possibility of privacy invasions, we predicted that the effect of autonomy on conspiracy beliefs would be stronger in the United States than in China (*Hypothesis 4*).

### Method

The experiments were conducted online in the United States (using Prolific) and China (using Credamo). We translated the manipulations and measurements into Chinese using the back‐translation method.

#### Participants

Using G*power 3.1 (Faul et al., 2009) for an ANOVA with Fixed effects, special, main effects and interactions, a sensitivity analysis indicated that with a total sample size of 800, with 80% power, a Type I error rate of 5%, and 4 groups (autonomy × country), we can detect an interaction with a small effect size (*f* = 0.10). In total, we recruited 797 participants^3^ (355 males, 434 females, and 8 other; mean age 35.56, *SD* = 12.39) across two samples, with approximately 100 participants assigned to each group.

#### Procedure and measures

##### Manipulations of autonomy and interdependence of AI

The materials of Study 2 were revised, translated into Chinese, and used in Study 4. Also, the manipulation check items of autonomy (*α*_US_ = .94; *α*_China_ = .94) and interdependence (*α*_US_ = .95; *α*_China_ = .97), conspiracy beliefs (*α*_US_ = .92; *α*_China_ = .95) and perceived threat to society (*α*_US_ = .97; *α*_China_ = .94) were the same as in Study 2.

##### Privacy concerns

Privacy concerns were measured using the 10‐item (*α*_US_ = .87; *α*_China_ = .76) scale of Internet Users' Information Privacy Concerns (Groß, 2021). An example item is ‘it usually bothers me when online companies ask me for personal information’ (1 = *completely disagree* to 7 = *completely agree*).

##### Control variables

At the end of the experiment, participants were asked to provide their information about gender and age.^4^

### Results

#### Measurement invariance analyses

We tested for configural, metric and scalar invariance using three nested models. The results suggested full measurement invariance for all scales (see Supporting Information for details).

#### Manipulation checks

Two 2 (autonomy: high vs. low) × 2 (interdependence: high vs. low) × 2 (country: United States vs. China) ANOVAs with control variables included as covariates were conducted on the manipulation check items. For the autonomy manipulation, the result indicated that the main effect of autonomy was significant and in the predicted direction, *p* < .001, *η* ^2^ = .262, see Table 5 for means and *SD*s, and see Table 6 for more information about the ANOVAs. The main effect of interdependence was non‐significant. The main effect of country was significant. Specifically, Chinese participants perceived the AI as more autonomous than US participants. Among the interaction effects, only the interaction between the autonomy manipulation and country was significant. The effect of the autonomy manipulation was stronger in China, *F*(1, 391) = 261.743, *p* < .001, *η* ^2^ = .401, 90% CI = [0.342, 0.455], than in the US, *F*(1, 393) = 69.451, *p* < .001, *η* ^2^ = .150, 90% CI = [0.100, 0.204].

**TABLE 5:** Numbers of participants, means and *SD*s of main variables in Study 4.

	Autonomy		Interdependence	
	Low	High	Low	High
	Mean (*SD*)			
Autonomy perception	2.99 (1.66)	4.92 (1.66)	3.98 (1.94)	3.91 (1.90)
Interdependence perception	4.27 (1.98)	3.92 (1.96)	2.76 (1.63)	5.47 (1.21)
Conspiracy beliefs	2.73 (1.54)	3.43 (1.69)	3.45 (1.73)	2.70 (1.47)
Threat to society	2.76 (1.70)	3.24 (1.88)	3.48 (1.96)	2.51 (1.49)
*N*	402	395	404	393

**TABLE 6:** ANOVA results in Study 4.

	*F*	*p*	*η* ^2^	90% CI
Outcome variable: autonomy perception				
Autonomy manipulation	279.167	<.001	.262	[0.221, 0.303]
Interdependence manipulation	1.006	.316	.001	[0.000, 0.009]
Country	19.905	<.001	.025	[0.010, 0.045]
Autonomy × country	14.917	<.001	.019	[0.006, 0.037]
Outcome variable: interdependence perception				
Autonomy manipulation	16.026	<.001	.020	[0.007, 0.039]
Interdependence manipulation	778.643	<.001	.498	[0.460, 0.532]
Country	9.243	.002	.012	[0.002, 0.027]
Autonomy × country	9.514	.002	.012	[0.003, 0.028]
Interdependence × country	38.711	<.001	.047	[0.026, 0.073]
Outcome variable: conspiracy beliefs				
Autonomy manipulation	43.521	<.001	.052	[0.030, 0.080]
Interdependence manipulation	49.772	<.001	.060	[0.036, 0.088]
Country	19.915	<.001	.025	[0.010, 0.045]
Autonomy × Interdependence	1.042	.308	.001	[0.000, 0.009]
Autonomy × country	1.708	.192	.002	[0.000, 0.011]
Interdependence × country	13.261	<.001	.017	[0.005, 0.034]
Autonomy × interdependence × country	6.067	.014	.008	[0.001, 0.021]

*Note*: × refers to an interaction term. For *F*s, the degrees of freedom are (1, 786).

For perceived interdependence, the main effect of interdependence was significant and in the predicted direction, *p* < .001, *η* ^2^ = .498. The main effect of autonomy was significant, with lower interdependence in the high autonomy condition. The main effect of country was significant, as Chinese participants perceived the AI as more interdependent than US participants. The interaction between the interdependence manipulation and country was also significant. The effect of the interdependence manipulation was stronger in China, *F*(1, 391) = 723.723, *p* < .001, *η* ^2^ = .649, 90% CI = [0.608, 0.685], than in the US, *F*(1, 393) = 195.946, *p* < .001, *η* ^2^ = .333, 90% CI = [0.273, 0.389]. The interaction between the autonomy manipulation and country was also significant. The effect of the autonomy manipulation on interdependence perception was stronger in the United States, *F*(1, 393) = 20.945, *p* < .001, *η* ^2^ = .051, 90% CI = [0.021, 0.090], than in China, *F*(1, 391) = 0.530, *p* = .467, *η* ^2^ = .001, 90% CI = [0.000, 0.014].

#### Conspiracy beliefs

A 2 (autonomy: high vs. low) × 2 (interdependence: high vs. low) × 2 (country: United States vs. China) ANOVA with control variables was conducted. The main effect of autonomy on conspiracy beliefs was significant, supporting H1. The main effect of interdependence was also significant, supporting H2. The main effect of country was also significant, with US participants showing higher levels of conspiracy beliefs than Chinese participants.

The three‐way interaction effect among interdependence, autonomy, and country was significant. Unexpectedly, the interaction effect between interdependence and country was also significant. The effect of interdependence was weaker in the United States, *F*(1, 393) = 5.303, *p* = .022, *η* ^2^ = .013, 90% CI = [0.001, 0.038], than in China, *F*(1, 391) = 63.932, *p* < .001, *η* ^2^ = .141, 90% CI = [0.092, 0.194]. However, the interaction between autonomy and country was non‐significant, which does not support Hypothesis 4. The interaction effect between autonomy and interdependence was also non‐significant.

#### The mediating effect of perceived threat to society

**FIGURE 4:**
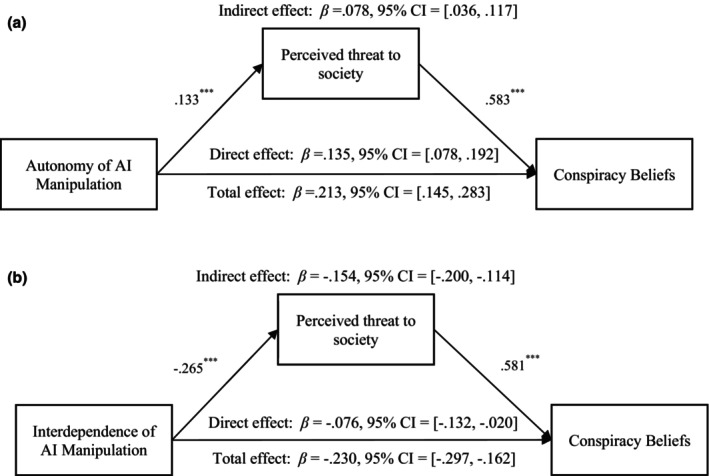
(a) The mediational effect of perceived threat to society on the relationship between autonomy manipulation and conspiracy beliefs. ***p < .001. Control variables (i.e. gender, age) were included. Autonomy of AI was coded as 1 for low autonomy and 2 for high autonomy. (b) The mediational effect of perceived threat to society on the relationship between interdependence manipulation and conspiracy beliefs. ***p < .001. Control variables (i.e. gender, age) were included. Interdependence of AI was coded as 1 for low interdependence and 2 for high interdependence.

The mediating effect of perceived threat to society between autonomy and conspiracy beliefs was significant, *β*_indirect effect_ = .078, *SE* = 0.021, *p* < .001, 95% CI = [0.036, 0.117] (see Figure 4a). The mediating effect of perceived threat to society between interdependence and conspiracy beliefs was also significant, *β*_indirect effect_ = −.154, *SE* = 0.022, *p* < .001, 95% CI = [−0.200, −0.114] (see Figure 4b). These findings support Hypotheses 3a and 3b.

#### Privacy concerns

For exploratory purposes, we examined why Chinese and American participants differed in their AI‐related conspiracy beliefs (full results are in Supporting Information). We first tested the mediating effect of privacy concern in the link between country and conspiracy beliefs. The mediating effect of privacy concern was significant, suggesting that compared to Americans, Chinese participants had lower AI‐related conspiracy beliefs due to lower privacy concerns.

### Discussion

In Study 4, we examined our hypotheses in US and Chinese samples. We again found evidence for the main effects of autonomy and interdependence (Hypotheses 1 and 2), and the mediating role of perceived threat to society (Hypotheses 3a and 3b). Somewhat exploratively, we also found that AI‐conspiracy beliefs were higher in the US than in China, which could be explained by the finding that the US participants were more concerned about their privacy than Chinese participants. No significant difference in the effect of autonomy on conspiracy beliefs emerged between the US and China, which did not support H4.

## MINI META‐ANALYSIS

Given the inconsistency in the mediating effects of perceived threat to society among previous studies, we conducted a mini meta‐analysis using data collected across Studies 1–4 to evaluate these effects, and Study 4 was divided into two samples based on participants' nationality.

### Method

We conducted this method for two models with different independent variables (i.e. autonomy and interdependence of AI), the same mediator (i.e. perceived threat to society) and a dependent variable (i.e. conspiracy beliefs). For these models, using two‐stage structural equation modelling (TSSEM; Cheung, 2022; Cheung, 2014), we first estimated an average correlation matrix with a random effects model in the first stage (see Supporting Information). Then we fitted the mediation model with the correlation matrix in the second stage.

### Results and Discussion

There was no meaningful statistic on the model fit since the model was saturated. For the model with autonomy as a predictor, the estimated indirect effect was significant, *β* = −.120, 95% CI = [−0.191, −0.057]. For the model with interdependence as a predictor, the estimated indirect effect was also significant, *B* = 0.084, 95% CI = [0.056, 0.116] (see Table 7 for details). The results confirmed Hypotheses 3a and 3b.

**TABLE 7:** Results of the second stage in mini meta‐analysis.

	*Β*	Lower bound	Upper bound
Model with Autonomy as predictor			
Autonomy → Threat	−0.211	−0.322	−0.101
Threat → Conspiracy Beliefs	0.568	0.488	0.649
Autonomy → Conspiracy Beliefs (direct effect)	−0.082	−0.168	0.011
Indirect effect	−0.120	−0.191	−0.057
Model with Interdependence as predictor			
Interdependence → Threat	0.150	0.101	0.199
Threat → Conspiracy Beliefs	0.562	0.483	0.640
Interdependence → Conspiracy Beliefs (direct effect)	0.156	0.116	0.195
Indirect effect	0.084	0.056	0.116

*Note*: X → Y represents the path coefficient from variable X to variable Y. Lower bound and upper bound represent the 95% CI.

## GENERAL DISCUSSION

The goal of the current research was to examine what features of AI would predict AI‐related conspiracy beliefs. We investigated this using a survey, three experiments and a mini meta‐analysis. Across studies, the results revealed that the perceived autonomy of AI increased conspiracy beliefs (H1), while the perceived interdependence of AI decreased conspiracy beliefs (H2). In addition, the mediating role of perceived threat to society in the relationship between autonomy (H3a) or interdependence (H3b) and conspiracy beliefs also received support in most studies. The results in Study 4 were robust across US and Chinese samples, which supports the generalizability of the findings. Although we did not find support for the moderating effect of country on the effect of autonomy on conspiracy beliefs (H4), we did find an unexpected moderating effect of country on the effect of interdependence. In addition, Chinese participants showed fewer conspiracy beliefs and more positive attitudes towards AI than US participants, which can be attributed to lower privacy concerns among Chinese participants.

### Implications

The current research contributes to the vastly expanding literature of conspiracy beliefs in at least two ways. First, our research is the first attempt to investigate predictors of conspiracy theories specifically in the context of AI. The current era includes quick technological progress, and AI is likely to become an increasingly integrated part of society in the future. This will inevitably inspire conspiracy theories which may decrease the trust in, and acceptance of, AI. Second, our study offers broader theoretical insights by highlighting the role of perceived threats as a mechanism explaining belief in conspiracy theories in the context of AI. This may also help generate novel predictions; for instance, features of new technology that people find threatening seem to elicit conspiracy theories.

The current findings also have implications for the emerging literature on psychological responses to AI. A growing list of studies has focused on the perceived risks or threats associated with AI (Nowak et al., 2018; Stein et al., 2019). From both a theoretical and practical perspective, it is useful to understand what exact features the public perceives as threatening, how these perceptions may be attenuated, and what may drive support for AI (Choung et al., 2023; Kelly et al., 2023). For instance, Złotowski et al. (2017) have argued that autonomous AI would increase the extent to which people consider AI threatening, and therefore decrease their acceptance of it—a notion also supported by the findings presented here. The current research contributes to these issues by showing that both the autonomy and interdependence of AI matter for people's trust in, and attitudes towards, AI and AI developers. In addition, the current findings could provide AI developers and its management with practical suggestions about the technical features of AI that should be prioritized in the development process to gain public support.

While the results identified in the current research replicated in China and the United States (Study 4), our results also suggested differences between cultures that are relevant for predicting the extent to which the public will accept AI. For instance, cultures differ in how concerned citizens are about privacy, and this matters for their attitudes towards AI. These findings are relevant given the significance of accepting the reality of AI throughout countries and cultures (Kelly et al., 2023). Future research could further examine the differential effects of various features of AI on public perception across cultures and settings and explore the possible underlying mechanisms.

### Limitations and future directions

The current research had its limitations. First, manipulation check findings indicated that the experimental manipulations were not completely orthogonal in Studies 2 and 4. This made it more difficult to interpret the unique effects of these different manipulations on the dependent variables. This concern was mitigated by the finding that the manipulations in Study 3 were found to be orthogonal; however, Study 3 results were mostly consistent with the results observed in Studies 2 and 4. Moreover, Study 1 was a correlational survey study, which also yielded consistent results. Altogether, the consistency of the main results instills confidence that the orthogonality of the manipulations did not preclude a correct interpretation of these results across studies.

Across studies, the main effects of autonomy and interdependence on conspiracy beliefs were consistent and significant. While the mediating role of perceived threat to society only received partial support in Studies 2 and 3, the results of the mini meta‐analysis confirmed this mediating role.

In the current experiments, we operationalized the threat posed by the autonomy of AI through issues related to privacy. Although privacy is a frequently raised issue that makes people hesitant about using AI (Posey et al., 2011; Schlund & Zitek, 2021), there may be other types of threats that high autonomy of AI poses, such as job replacement (Huang & Rust, 2018). Therefore, future research could examine whether the current findings generalize to other types of perceived threats.

Our work used fictional vignettes as stimulus materials, which may limit the generalizability of our findings. Future studies could consider using field experiments or actual interactions with AI to increase the ecological validity.

We did not explore other underlying mechanisms in our studies. Threat perceptions might not be the only explanation of the main effects. For instance, perceived high autonomy of AI might invoke users' motivations to find explanations for AI's ambiguous behaviours. Furthermore, AI‐related conspiracy theories could provide (easy) explanations to users that attract them. Also, low interdependence of AI might lead to distrust towards institutions, and in turn, generate AI‐related conspiracy beliefs. Another potential mediator is perceived anthropomorphism of AI (Letheren et al., 2021): Autonomy or perceived interdependence manipulations are likely to influence the extent to which users consider AI as human‐like (i.e. anthropomorphism), which might influence their acceptance of AI‐related conspiracy theories, particularly those that portray AI as the conspirator. These issues suggest ample opportunities for future studies to explore the underlying mechanisms explaining AI‐related conspiracy beliefs.

In addition, our data were collected online, suggesting that the participants in the current studies had at least some basic level of digital literacy. As such, these participants are likely to be familiar with AI, possibly more so than citizens with lower levels of digital literacy. The subjective experience with, and prior opinions about, AI might influence their responses towards experimental materials. These considerations suggest that it might be worthwhile to also collect offline data, covering more diverse samples, in future studies.

Finally, we did not measure other conspiracy measures, for example, conspiracy mentality, in the current studies. Although our scale was reliable, had good construct validity and was related to the independent variables in predictable ways, this does prevent us from further validating the AI‐related conspiracy belief scale in this research. While the current results are promising, we recommend future studies to validate this scale further.

## CONCLUSIONS

In recent years, AI has developed rapidly. While the technology associated with AI may be remarkable and provide a high potential to improve human societies, this does not mean that the public will accept AI unconditionally. Indeed, many people are quite suspicious of AI, and AI‐related conspiracy theories have gradually emerged. In the current study, our findings suggested that perceived autonomy and interdependence matter for the extent to which people find AI threatening and their AI‐related conspiracy beliefs. These findings may inform the question of what scientists, technological developers and policymakers can do to effectively integrate AI in people's lives and reduce beliefs that place AI against the greater good of humanity.

## AUTHOR CONTRIBUTIONS

**Qi Zhao:** Conceptualization; data curation; formal analysis; funding acquisition; investigation; methodology; project administration; resources; writing – original draft; writing – review and editing. **Jan‐Willem van Prooijen:** Conceptualization; methodology; resources; supervision; writing – review and editing. **Xinying Jiang:** Conceptualization; methodology; resources; writing – review and editing. **Giuliana Spadaro:** Conceptualization; supervision; writing – review and editing.

## FUNDING INFORMATION

Our work was funded by a scholarship from the China Scholarship Council (grant no. 202206040003).

## CONFLICT OF INTEREST STATEMENT

We have no known conflict of interest to disclose.

The current paper is not under consideration or in press elsewhere.

## ETHICAL APPROVAL

This study was approved by the Scientific and Ethical Review Board (VCWE) of the Faculty of Behavior & Movement Sciences, VU Amsterdam. Approval number: VCWE‐S‐21‐00228.

## Supporting information


Appendix S1.

## Data Availability

The designs, materials and analysis plans for the experimental Studies 3 and 4 were pre‐registered at As Predicted (Study 3: https://aspredicted.org/NBD_B8T; Study 4: https://aspredicted.org/RCK_LY9). The data and analysis scripts for all studies have been made publicly available via OSF and can be accessed at https://osf.io/8jt5s. Full materials for all studies are available in the Supporting Information.

## Footnotes

^1^
The manipulation materials of Studies 2, 3 and 4 can be found in Supporting Information.

^2^
One participant reported an age of 7410, which we treated as missing information. The results are similar including this participant.

^3^
Because of technical error in this study, two participants did not give consent but completed the survey. We excluded them from analyses. One participant reported an age of 556, which we treated as missing information. Results were similar with this participant.

^4^
We asked for participants' educational level and political orientation in the US sample and participants' educational level in the Chinese sample. Because the levels of the variable education in United States and China are non‐comparable, we did not add this variable as a covariate.
