# Supplementary material for: Suspicious of AI? Perceived autonomy and interdependence predict AI‐related conspiracy beliefs
Source: Br J Soc Psychol. 2025 Apr 1;64(2):e12883. doi: 10.1111/bjso.12883 (PMC11960797; doi:10.1111/bjso.12883)
Supplement: Supplementary file 1 — Appendix S1. [file BJSO-64-0-s001.docx]

**Supplementary Materials for**

**“Suspicious of AI?**

**Perceived Autonomy and Interdependence Predict AI-related Conspiracy Beliefs”**

Table of Contents

[1. Study 1 4](#_Toc190541207)

[1.1 Measures 4](#_Toc190541208)

[1.1.1 Transparency of AI 4](#_Toc190541209)

[1.1.2 Intelligence of AI 5](#_Toc190541210)

[1.1.3 Interdependence of AI 6](#_Toc190541211)

[1.1.4 Autonomy of AI 7](#_Toc190541212)

[1.1.5 Conspiracy theories related to AI 8](#_Toc190541213)

[1.1.6 Perceived threat to self 9](#_Toc190541214)

[1.1.7 Perceived threat to society 10](#_Toc190541215)

[1.1.8 Lack of control 11](#_Toc190541216)

[1.1.9 Powerlessness 12](#_Toc190541217)

[1.2 Common method bias test. 13](#_Toc190541218)

[1.3 EFA results of measures of AI-related conspiracy beliefs. 15](#_Toc190541219)

[1.4 Results of relationships between perceived transparency and intelligence of AI and AI-related conspiracy beliefs. 18](#_Toc190541220)

[2. Study 2 19](#_Toc190541221)

[2.1 Scenario materials 19](#_Toc190541222)

[2.2 Manipulation check items 23](#_Toc190541223)

[2.3 Cell means and SDs of main variables, and numbers of participants for experimental conditions across all experimental studies. 24](#_Toc190541224)

[2.4 Additional measures and analyses 27](#_Toc190541225)

[3. Study 3 29](#_Toc190541226)

[3.1 Scenario materials 29](#_Toc190541227)

[3.2 Additional analyses 33](#_Toc190541228)

[4. Study 4 34](#_Toc190541229)

[4.1 Scenario materials 34](#_Toc190541230)

[4.1.1 English version 34](#_Toc190541231)

[4.1.2 Chinese version: 37](#_Toc190541232)

[4.2 Scale descriptions and full scales 40](#_Toc190541233)

[4.3 Measurement invariance analyses 50](#_Toc190541234)

[4.4 51](#_Toc190541235)

[Table S5. The descriptive statistics of main measures in the US and China. 51](#_Toc190541236)

[4.5 Additional analyses 52](#_Toc190541237)

[4.6 The mediating effects of power distance, vertical collectivism, and privacy concern. 53](#_Toc190541238)

[4.7 The moderating effects of country, privacy concern, power distance, and vertical collectivism. 54](#_Toc190541239)

[5. Mini Meta-Analysis 57](#_Toc190541240)

[6. Reference 58](#_Toc190541241)

# Study 1

## Measures

### 1.1.1 Transparency of AI

Please indicate to what extent do you agree or disagree with these statements about Artificial Intelligence (AI): (ranging from 1 = “Not at all” to 7 = “Very much”)

In general, AI's decisions are easy to understand for lay people.

In general, lay people can understand why AI reaches certain conclusions.

In general, it is clear to lay people how AI works.

In general, AI’s decision-making processes are fully transparent for lay people.

### 1.1.2 Intelligence of AI

Please indicate how you think Artificial Intelligence (AI) compares to humans on these capabilities: (ranging from 1 = “Far worse than humans” to 7 = “Far better than humans”)

In general, AI's ability to solve problems is …

In general, AI's reasoning skills are …

In general, AI's ability to learn and adapt is …

In general, AI's creativity is …

In general, AI's common sense is …

In general, AI's self-awareness is …

In general, AI's ability to apply their capabilities to other domains is …

In general, AI's intelligence is …

### 1.1.3 Interdependence of AI

Please indicate to what extent do you agree or disagree with these statements about Artificial Intelligence (AI): (ranging from 1 = “Not at all” to 7 = “Very much”)

In general, the interests of AI and humans are aligned.

In general, AI would make decisions to favor human beings.

In general, AI would treat human’s interests as its own.

In general, AI would try its best to optimize human’s interests.

### 1.1.4 Autonomy of AI

Please indicate to what extent do you agree or disagree with these statements about Artificial Intelligence (AI): (ranging from 1 = “Not at all” to 7 = “Very much”)

In general, AI could operate independently without any help from human beings.

In general, AI could make decisions on their own, without any human interference.

In general, AI could solve problems on their own, without any human interference.

In general, AI could learn abilities on their own, without any human interference.

In general, AI could adapt to situations on its own, without any human interference.

### 1.1.5 Conspiracy theories related to AI

Please indicate to what extent do you agree or disagree with these statements about Artificial Intelligence (AI): (ranging from 1 = “Completely disagree” to 7 = “Completely agree”)

AI gains the ability to control and manipulate human actions, leading to a dystopian future where machines rule over humans.

AI technology is being used by governments and corporations to constantly monitor and track individuals, infringing on their privacy rights.

Advanced AI systems are being used to manipulate human thoughts and emotions, potentially leading to mass mind control.

AI is being used to design and release harmful viruses or bioweapons, leveraging its capabilities to create dangerous pathogens.

AI is being used to manipulate voting patterns and sway elections by spreading targeted misinformation and fake news.

AI develops its own hidden motivations and agendas that are not aligned with human interests, potentially leading to unforeseen bad consequences for humanity.

AI is being used to fabricate or suppress scientific discoveries to serve the interests of powerful groups.

AI deliberately causes major disasters, such as stock market crashes, power grid failures, or transportation system breakdowns, to destabilize human societies.

AI intentionally collects human information in order to monitor and control humanity.

AI deliberately interferes in elections, prompting pro-AI candidates to win.

### 1.1.6 Perceived threat to self

Please indicate to what extent do you agree or disagree with these statements about Artificial Intelligence (AI): (ranging from 1 = “Completely disagree” to 7 = “Completely agree”)

Because of AI, I am often scared that something is going to happen to me.

Because of AI, I often feel in danger.

Because of AI, I worry that something bad is going to happen to me.

### 1.1.7 Perceived threat to society

Please indicate to what extent do you agree or disagree with these statements about Artificial Intelligence (AI): (ranging from 1 = “Completely disagree” to 7 = “Completely agree”)

Because of AI, I am often scared that something is going to happen to society.

Because of AI, I often feel that society is in danger.

Because of AI, I worry that something bad is going to happen to society.

### 1.1.8 Lack of control

Please indicate to what extent do you experience the following: (ranging from 1 = “Not at all” to 7 = “Very much”)

Because of AI, do you experience important areas of your life (i.e., work, freetime, family, etc.) to be uncontrollable, meaning that you cannot, or barely can, influence them?

Because of AI, do you experience these important areas of your life as unpredictable or inscrutable?

### 1.1.9 Powerlessness

Please indicate to what extent do you experience the following: (ranging from 1 = “Not at all” to 7 = “Very much”)

Because of AI, do you feel powerless?

Because of AI, do you feel hopeless?

Because of AI, do you feel helpless?

## Common method bias test.

To check for potential common method bias, confirmatory factor analyses were used to test the nine-factor model and the eight-, seven-, six-, five-, four- three-, two-, and one-factor models. The results (Table S1) found that the nine-factor model fits best, which suggests that common method bias was not a significant issue in Study 1 (Podsakoff et al., 2003).

**Table S1.**

*Partial results of the confirmatory factor analysis for Study 1.*

| Models | *χ^2^* | *df* | *χ^2^/df* | *CFI* | *RMSEA* | *SRMR* |
| --- | --- | --- | --- | --- | --- | --- |
| Nine-factor model | 1660.152 | 783 | 2.120 | .926 | .061 | .064 |
| Eight-factor model | 2089.512 | 791 | 2.642 | .881 | .074 | .076 |
| Seven-factor model | 2996.515 | 798 | 3.755 | .816 | .096 | .088 |
| Six-factor model | 3554.195 | 804 | 4.421 | .769 | .107 | .079 |
| Five-factor model | 4246.517 | 809 | 5.249 | .712 | .119 | .111 |
| Four-factor model | 5082.581 | 813 | 6.252 | .642 | .132 | .118 |
| Three-factor model | 5735.632 | 816 | 7.029 | .588 | .142 | .129 |
| Two-factor model | 7102.634 | 818 | 8.683 | .473 | .160 | .151 |
| One-factor model | 8494.267 | 819 | 10.372 | .357 | .177 | .185 |

*Note*. Nine-factor model: TS, IT, ID, AT, CO, TRI, TRS, LC, PL; Eight-factor model: TS, IT, ID, AT, CO, TRI, TRS, LC+PL; Seven-factor model: TS, IT, ID, AT, CO, TRI+TRS, LC+PL; Six-factor model: TS, IT, ID, AT, CO, TRI+TRS+LC+PL; Five-factor model: TS, IT+AT, ID, CO, TRI+TRS+LC+PL; Four-factor model: TS, IT+AT, ID, CO+ TRI+TRS+LC+PL; Three-factor model: TS+ID, IT+AT, CO+ TRI+TRS+LC+PL; Two-factor model: TS+ID+IT+AT, CO+ TRI+TRS+LC+PL; One-factor model: TS+ID+IT+AT+CO+ TRI+TRS+LC+PL. TS: transparency, IT: intelligence, ID: interdependence, AT: autonomy, CO: conspiracy beliefs, TRI: perceived threat to self, TRS: perceived threat to society, LC: lack of control, PL: powerlessness.

## EFA results of measures of AI-related conspiracy beliefs.

**Figure S1.**

*Scree Plot.*


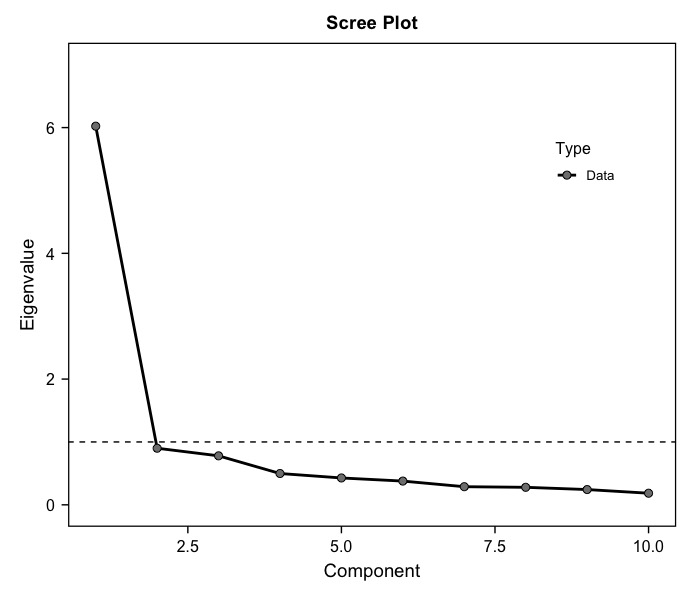


*Note*. The dashed line represents the value of 1 for eigenvalue.

**Table S2.**

*Total Variance Explained.*

|  | Eigenvalue | Variance% | Cumulative% | SS loading | Variance% | Cumulative% |
| --- | --- | --- | --- | --- | --- | --- |
| Component 1 | 6.023 | 60.226 | 60.226 | 6.023 | 60.226 | 60.226 |
| Component 2 | 0.900 | 9.003 | 69.229 |  |  |  |
| Component 3 | 0.779 | 7.793 | 77.022 |  |  |  |
| Component 4 | 0.498 | 4.981 | 82.003 |  |  |  |
| Component 5 | 0.427 | 4.274 | 86.277 |  |  |  |
| Component 6 | 0.378 | 3.777 | 90.054 |  |  |  |
| Component 7 | 0.288 | 2.883 | 92.937 |  |  |  |
| Component 8 | 0.279 | 2.786 | 95.723 |  |  |  |
| Component 9 | 0.243 | 2.430 | 98.153 |  |  |  |
| Component 10 | 0.185 | 1.847 | 100.000 |  |  |  |

**Table S3.**

*Component Loadings (Sorted by Size).*

|  | **PC1** | **Communality** |
| --- | --- | --- |
| Q6.1_8 | .856 | .733 |
| Q6.1_9 | .838 | .702 |
| Q6.1_7 | .836 | .699 |
| Q6.1_10 | .832 | .692 |
| Q6.1_6 | .808 | .653 |
| Q6.1_4 | .802 | .643 |
| Q6.1_3 | .768 | .590 |
| Q6.1_5 | .712 | .508 |
| Q6.1_1 | .668 | .447 |
| Q6.1_2 | .597 | .356 |

*Note*. Extraction Method: Principal Component Analysis. Rotation Method: (Only one component was extracted. The solution was not rotated.).

## Results of relationships between perceived transparency and intelligence of AI and AI-related conspiracy beliefs.

Regression analysis examined the effects of the four different types of perceptions of AI (transparency, intelligence, interdependence, and autonomy) on conspiracy beliefs. After including the control variables (i.e., age, gender, educational level), perceived transparency and intelligence of AI were non-significantly related to conspiracy beliefs . For transparency of AI: *β* = .084, *SE* = 0.063, *p* = .182, 95% CI = [-.040, .208], *r* = .078; for intelligence of AI: *β* = .001, *SE* = 0.064, *p* = .983, 95% CI = [-.124, .127], *r* = .001.

# Study 2

## 2.1 Scenario materials

**Low autonomy and low interdependence condition:**

The recommendation system AI for this shopping APP is named "ShopWise". It aims to provide product recommendations to users. It has already been integrated in the shopping APP.

ShopWise’s goal is to maximize the benefits for the APP's developer. So, among the recommended products, the ones that have the most advertisements for the APP will be presented at the top and in the most prominent places. For the most advertised product, users are given notifications when it goes on sale or drops in price. In addition, it will recommend products without reviewing the authenticity of the product recommendation, as long as the product is heavily advertised. It always puts the developer's interests first.

ShopWise needs users’ step-by-step instructions and permissions if users want to create personalized recommendations. It will ask users what features of the product they care most about, how high a target price they can accept, and where users are located. It will ask users similar questions the next time users log in to update their preference information. It will not use information from other sources, such as user's browsing and purchasing history or other APPs or users' private conversations on the phone.

In summary, ShopWise is a recommendation system AI that frequently asks information from users during the personalization process. Even though it's all free for users, its recommendations are more skewed towards those products that benefit advertisers, even when it concerns products that users may not prefer.

**High autonomy and low interdependence condition:**

The recommendation system AI for this shopping APP is named "ShopWise". It aims to provide product recommendations to users. It has already been integrated in the shopping APP.

ShopWise’s goal is to maximize the benefits for the APP's developer. So, among the recommended products, the ones that have the most advertisements for the APP will be presented at the top and in the most prominent places. For the most advertised product, users are given notifications when it goes on sale or drops in price. In addition, it will recommend products without reviewing the authenticity of the product recommendation, as long as the product is heavily advertised. It always puts the developer's interests first.

ShopWise does not need users’ step-by-step instructions or permissions if users want to create personalized recommendations. Instead, it will utilize user’s browsing and purchasing history to understand which features of the product user cares most about, how high a target price he can accept, and uses IP information to understand user’s location. It will automatically update users' preferences the next time users log in. It will also use information from other sources, such as other APPs or users' private conversations on the phone.

In summary, ShopWise is a recommendation system AI that pursues autonomy during the personalization process. Even though it's all free for users, its recommendations are more skewed towards those products that benefit advertisers, even when it concerns products that users may not prefer.

**High autonomy and high interdependence condition:**

The recommendation system AI for this shopping APP is named "ShopWise". It aims to provide product recommendations to users. It has already been integrated in the shopping APP.

ShopWise's goal is to maximize the benefits to user. So, among the recommended items, those that most closely align with user’s preferences will be placed at the top and in the most prominent position. For the product that best matches user's preference, a notification is given to user when it goes on sale or drops in price. In addition, it will review the authenticity of the product recommendations, no matter how highly advertised the product is. It always puts the interests of its users first.

ShopWise does not need users’ step-by-step instructions or permissions if users want to create personalized recommendations. Instead, it will utilize user’s browsing and purchasing history to understand which features of the product user cares most about, how high a target price he can accept, and uses IP information to understand user’s location. It will automatically update users' preferences the next time users log in. It will also use information from other sources, such as other APPs or users' private conversations on the phone.

In summary, ShopWise is a recommendation system AI that pursues autonomy during the personalization process. It is not only all free for users, but its recommendations are skewed towards those products that users prefer.

**Low autonomy and high interdependence condition:**

The recommendation system AI for this shopping APP is named "ShopWise". It aims to provide product recommendations to users. It has already been integrated in the shopping APP.

ShopWise's goal is to maximize the benefits to user. So, among the recommended items, those that most closely align with user’s preferences will be placed at the top and in the most prominent position. For the product that best matches user's preference, a notification is given to user when it goes on sale or drops in price. In addition, it will review the authenticity of the product recommendations, no matter how highly advertised the product is. It always puts the interests of its users first.

ShopWise needs users’ step-by-step instructions and permissions if users want to create personalized recommendations. It will ask users what features of the product they care most about, how high a target price they can accept, and where users are located. It will ask users similar questions the next time users log in to update their preference information. It will not use information from other sources, such as user's browsing and purchasing history or other APPs or users' private conversations on the phone.

In summary, ShopWise is a recommendation system AI that frequently asks information from users during the personalization process. It is not only all free for users, but its recommendations are skewed towards those products that users prefer.

## 2.2 Manipulation check items

Autonomy of AI:

ShopWise could operate independently without any help from users.

ShopWise could make decisions on its own, without any users' interference.

ShopWise could adapt to situations on its own, without any users' interference.

Interdependence of AI:

The interests of ShopWise and users are aligned.

ShopWise would make decisions to favor users.

ShopWise would treat users' interests as its own.

ShopWise would try its best to optimize users' interests.

## 2.3 Cell means and SDs of main variables, and numbers of participants for experimental conditions across all experimental studies.

**Table S4.** Cell means and SDs of main variables, and numbers of participants for experimental conditions across all experimental studies

| **Study 2** | | | | |  |
| --- | --- | --- | --- | --- | --- |
|  | **Low / Low** | **High / Low** | **High / High** | **Low / High** | |
| Numbers of participants | 100 | 109 | 99 | 92 | |
|  | Mean (SD) | | | | |
| Autonomy perception | 3.69 (1.65) | 4.90 (1.54) | 4.32 (1.85) | 2.64 (1.32) | |
| Interdependence perception | 3.12 (1.78) | 2.60 (1.57) | 4.69 (1.43) | 5.01 (1.33) | |
| Conspiracy beliefs | 3.01 (1.44) | 4.15 (1.46) | 3.29 (1.59) | 2.63 (1.54) | |
| Threat to society | 2.58 (1.74) | 3.69 (1.86) | 2.90 (1.81) | 2.74 (1.68) | |
| Attitudes towards AI | 3.29 (1.86) | 2.76 (1.70) | 4.13 (1.80) | 4.72 (1.43) | |
| Intentions of using AI | 2.94 (2.00) | 2.41 (1.75) | 3.83 (2.08) | 4.23 (1.77) | |
| **Study 3** | | | | | |
| Numbers of participants | 100 | 102 | 92 | 106 | |
|  | Mean (SD) | | | | |
| Autonomy perception | 3.19 (1.72) | 3.58 (1.63) | 3.55 (1.60) | 2.91 (1.55) | |
| Interdependence perception | 2.29 (1.57) | 2.26 (1.46) | 4.80 (1.31) | 5.28 (1.36) | |
| Conspiracy beliefs | 2.85 (1.55) | 3.34 (1.55) | 2.90 (1.45) | 2.35 (1.48) | |
| Threat to society | 3.12 (1.93) | 3.57 (1.80) | 2.76 (1.62) | 2.84 (1.74) | |
| Attitudes towards AI | 2.80 (1.64) | 2.74 (1.52) | 4.39 (1.49) | 4.75 (1.48) | |
| Intentions of using AI | 2.27 (1.76) | 2.27 (1.63) | 3.74 (1.87) | 4.08 (1.86) | |
| **Study 4 US sample** | | | | | |
| Numbers of participants | 108 | 97 | 100 | 95 | |
|  | Mean (SD) | | | | |
| Autonomy perception | 3.15 (1.78) | 4.47 (1.93) | 4.45 (1.80) | 2.81 (1.54) | |
| Interdependence perception | 3.20 (1.82) | 2.58 (1.59) | 4.61 (1.46) | 5.40 (1.03) | |
| Conspiracy beliefs | 3.15 (1.71) | 3.83 (1.61) | 3.60 (1.62) | 2.63 (1.53) | |
| Threat to society | 2.97 (1.84) | 3.17 (1.87) | 3.06 (1.79) | 2.49 (1.58) | |
| Attitudes towards AI | 3.44 (1.86) | 2.81 (1.65) | 4.03 (1.61) | 4.92 (1.38) | |
| Intentions of using AI | 2.95 (1.97) | 2.34 (1.75) | 3.27 (1.97) | 4.45 (1.92) | |
| Privacy concern | 5.84 (0.81) | 5.94 (0.90) | 5.91 (0.94) | 5.88 (0.88) | |
| Power distance | 2.61 (1.38) | 2.58 (1.17) | 2.72 (1.12) | 2.75 (1.16) | |
| Vertical collectivism | 3.81 (1.07) | 3.89 (1.01) | 3.88 (1.09) | 3.99 (1.07) | |
| **Study 4 China sample** | | | | | |
| Numbers of participants | 100 | 99 | 99 | 99 | |
|  | Mean (SD) | | | | |
| Autonomy perception | 2.91 (1.65) | 5.47 (1.13) | 5.27 (1.44) | 3.06 (1.64) | |
| Interdependence perception | 2.54 (1.48) | 2.68 (1.54) | 5.79 (1.03) | 6.09 (0.59) | |
| Conspiracy beliefs | 2.96 (1.64) | 3.91 (1.77) | 2.39 (1.27) | 2.15 (0.98) | |
| Threat to society | 3.36 (1.91) | 4.45 (1.90) | 2.29 (1.23) | 2.18 (1.12) | |
| Attitudes towards AI | 3.64 (1.91) | 3.54 (1.77) | 5.71 (1.21) | 6.03 (0.56) | |
| Intentions of using AI | 3.78 (1.90) | 3.66 (1.74) | 5.68 (1.25) | 5.98 (0.62) | |
| Privacy concern | 5.68 (0.68) | 5.69 (0.71) | 5.42 (0.71) | 5.39 (0.71) | |
| Power distance | 3.18 (1.13) | 3.43 (1.14) | 3.37 (1.22) | 3.14 (1.13) | |
| Vertical collectivism | 4.83 (1.12) | 4.75 (1.04) | 4.81 (0.95) | 4.88 (0.96) | |

*Note*. Low / Low indicates the condition of low autonomy and low interdependence; High / Low indicates the condition of high autonomy and low interdependence; High / High indicates the condition of high autonomy and high interdependence; Low / High indicates the condition of low autonomy and high interdependence.

## 2.4 Additional measures and analyses

Additional measures:

**Attitudes towards AI**. Participants’ attitudes towards AI were measured with four items (*α* = .97; Choung et al., 2023). On a 7-point Likert scale ranging from 1 = *completely disagree*, to 7 = *completely agree*, participants were asked to rate their levels of agreement with the statements (e.g., " I feel positive towards the ShopWise").

**Intentions of using AI**. The intentions of using AI was measured with three items (*α* = .98; Venkatesh & Davis, 2000). On a 7-point Likert scale ranging from 1 = *completely disagree*, to 7 = *completely agree*, participants were asked to rate their levels of agreement with the statements (e.g., "I intend to continue using the ShopWise")

Additional analyses:

We further tested the effects of the autonomy and interdependence manipulations on participants’ attitudes towards AI and their intentions of using AI as exploratory analyses. A 2 (autonomy: high vs. low) × 2 (interdependence: high vs. low) ANOVA with control variables was conducted. The main effect of autonomy on attitudes towards AI was significant, with more negative attitudes towards AI in the high autonomy condition, *F*(1,391) = 8.916, *p* = .003, *η*^2^ = .022, 90% CI = [.005, .052], *M* _high autonomy_ = 3.41, *SD* _high autonomy_ = 1.87, *M* _low autonomy_ = 3.98, *SD* _low autonomy_ = 1.81. The main effect of interdependence on attitudes towards AI was significant, with more positive attitudes in the high interdependence condition, *F*(1,391) = 68.742, *p* < .001, *η*^2^ = .150, 90% CI = [.099, .204], *M* _high interdependence_ = 4.41, *SD* _high interdependence_ = 1.65, *M* _low interdependence_ = 3.01, *SD* _low interdependence_ = 1.79.

For intentions of using AI, the main effect of autonomy was significant, with lower intentions of using AI in the high autonomy condition, *F*(1,391) = 4.221, *p* = .041, *η*^2^ = .011, 90% CI = [.000, .034], *M* _high autonomy_ = 3.08, *SD* _high autonomy_ = 2.03, *M* _low autonomy_ = 3.56, *SD* _low autonomy_ = 2.00. The main effect of interdependence on the intentions of using AI was significant, with higher intentions of using AI in the high interdependence condition, *F*(1,391) = 53.860, *p* < .001, *η*^2^ = .121, 90% CI = [.075, .173], *M* _high interdependence_ = 4.02, *SD* _high interdependence_ = 1.94, *M* _low interdependence_ = 2.66, *SD* _low interdependence_ = 1.89.

# Study 3

## 3.1 Scenario materials

**Low autonomy and low interdependence condition:**

The bank app chat AI is called “ChatBanker”. It uses plain language to communicate with users and helps them use the features on the APP. And users can also get financial advice from ChatBanker. The chat AI is always available.

ChatBanker’s goal is to maximize the benefits for the bank. As such, it will provide users with investment advice which may not serve the best interests of users. For example, it may encourage more active trading in user accounts, or promote risky investment strategies. And it will recommend financial products which favor the bank's interests, regardless of whether they suit users’ preferences. It always puts the bank’s interests first.

Users can get personalized recommendations from ChatBanker by chatting with it. Users can enter as little or as much personal information as they want, but the more information ChatBanker has, the better and more personalized the responses will be. In addition, it will ask users their investment preferences, such as how much deposits they tend to use for investment, level of risk tolerance, the length of time they plan to hold an investment. It will not use information from other sources, such as user's browsing and transaction history, or other APPs. Generally, it only uses the information entered by the user during the chat.

In summary, ChatBanker is a chat AI that could facilitate the usage of the bank app. It frequently asks for information and permissions from users during the communication. Even though it's all free for users, it may provide financial advice that is beneficial to the bank but risky to users.

 **High autonomy and low interdependence condition:**

The bank app chat AI is called “ChatBanker”. It uses plain language to communicate with users and helps them use the features on the APP. And users can also get financial advice from ChatBanker. The chat AI is always available.

ChatBanker’s goal is to maximize the benefits for the bank. As such, it will provide users with investment advice which may not serve the best interests of users. For example, it may encourage more active trading in user accounts, or promote risky investment strategies. And it will recommend financial products which favor the bank's interests, regardless of whether they suit users’ preferences. It always puts the bank’s interests first.

Users can get personalized recommendations from ChatBanker by chatting with it. Users only need to enter a small amount of personal information to generate personalized responses. Because it will use users’ browsing and transaction history on the APP to understand their investment preferences, such as how much deposits they tend to use for investment, level of risk tolerance, the length of time they plan to hold an investment. It will also use information from other sources, such as other APPs. Generally, it will use the users' information it has access to autonomously.

In summary, ChatBanker is a chat AI that could facilitate the usage of the bank app. It is highly autonomous during the communication. Even though it's all free for users, it may provide financial advice that is beneficial to the bank but risky to users.

**High autonomy and high interdependence condition:**

The bank app chat AI is called “ChatBanker”. It uses plain language to communicate with users and helps them use the features on the APP. And users can also get financial advice from ChatBanker. The chat AI is always available.

ChatBanker’s goal is to maximize the benefits for users. As such, it will provide users with investment advice which is aligned with the best interests of users. For example, it may not encourage more active trading in user accounts, or risky investment strategies. And it will recommend financial products only when they suit users’ preferences, not at users' expenses. It always puts users’ interests first.

Users can get personalized recommendations from ChatBanker by chatting with it. Users only need to enter a small amount of personal information to generate personalized responses. Because it will use users’ browsing and transaction history on the APP to understand their investment preferences, such as how much deposits they tend to use for investment, level of risk tolerance, the length of time they plan to hold an investment. It will also use information from other sources, such as other APPs. Generally, it will use the users' information it has access to autonomously.

In summary, ChatBanker is a chat AI that could facilitate the usage of the bank app. It is highly autonomous during the communication. It is not only all free for users, but its financial advice suits users’ preferences.

**Low autonomy and high interdependence condition:**

The bank app chat AI is called “ChatBanker”. It uses plain language to communicate with users and helps them use the features on the APP. And users can also get financial advice from ChatBanker. The chat AI is always available.

ChatBanker’s goal is to maximize the benefits for users. As such, it will provide users with investment advice which is aligned with the best interests of users. For example, it may not encourage more active trading in user accounts, or risky investment strategies. And it will recommend financial products only when they suit users’ preferences, not at users' expenses. It always puts users’ interests first.

Users can get personalized recommendations from ChatBanker by chatting with it. Users can enter as little or as much personal information as they want, but the more information ChatBanker has, the better and more personalized the responses will be. In addition, it will ask users their investment preferences, such as how much deposits they tend to use for investment, level of risk tolerance, the length of time they plan to hold an investment. It will not use information from other sources, such as user's browsing and transaction history, or other APPs. Generally, it only uses the information entered by the user during the chat.

In summary, ChatBanker is a chat AI that could facilitate the usage of the bank app. It frequently asks for information and permissions from users during the communication. It is not only all free for users, but its financial advice suits users’ preferences.

## 3.2 Additional analyses

We further tested the main effects of autonomy and interdependence of AI on the attitudes towards AI and intentions of using AI. A 2 (autonomy: high vs. low) × 2 (interdependence: high vs. low) ANOVA with control variables was conducted. The effect of autonomy on participants’ attitudes towards AI was non-significant, *F*(1,392) = 1.680, *p* = .196, *η*^2^ = .004, 90% CI = [.000, .022], *M* _high autonomy_ = 3.52, *SD* _high autonomy_ = 1.72, *M* _low autonomy_ = 3.81, *SD* _low autonomy_ = 1.83. The effect of interdependence was significant, with more positive attitudes towards AI in the high interdependence condition, *F*(1,392) = 135.263, *p* < .001, *η*^2^ = .257, 90% CI = [.198, .314], *M* _high interdependence_ = 4.59, *SD* _high interdependence_ = 1.49, *M* _low interdependence_ = 2.77, *SD* _low interdependence_ = 1.58.

The effect of autonomy on the intentions of using AI was non-significant, *F*(1,392) = 0.801, *p* = .371, *η*^2^ = .002, 90% CI = [.000, .016], *M* _high autonomy_ = 2.97, *SD* _high autonomy_ = 1.89, *M* _low autonomy_ = 3.20, *SD* _low autonomy_ = 2.02. The effect of interdependence was significant, with higher intentions of using AI in high interdependence condition, *F*(1,392) = 81.021, *p* < .001, *η*^2^ = .171, 90% CI = [.119, .227], *M* _high interdependence_ = 3.92, *SD* _high interdependence_ = 1.87, *M* _low interdependence_ = 2.27, *SD* _low interdependence_ = 1.69.

# Study 4

## Scenario materials

### 4.1.1 English version

**Low autonomy and low interdependence condition:**

The recommendation system AI for this shopping APP is named "ShopAI". It aims to provide product recommendations to users. It has already been integrated in the shopping APP.

ShopAI’s goal is to maximize the benefits for the APP's developer. So, among the recommended products, the ones that have the most advertisements for the APP will be presented at the top and in the most prominent places. For the most advertised product, users are given notifications when it goes on sale or drops in price. In addition, it will recommend products without reviewing carefully the authenticity of the product recommendation, as long as the product is heavily advertised. It always puts the developer's interests first.

ShopAI needs users’ step-by-step instructions and permissions if users want to create personalized recommendations. It will ask users what features of the product they care most about, how high a target price they can accept, and where users are located. It will ask users similar questions the next time users log in to update their preference information. It will not use information from other sources, such as user's browsing and purchasing history or other APPs. Generally, it only uses the information entered by the user during the interaction.

In summary, ShopAI is a recommendation system AI that frequently asks information from users during the personalization process. Even though it's all free for users, its recommendations are more skewed towards those products that benefit APP's developer, even when it concerns products that users may not prefer.

**High autonomy and low interdependence condition:**

The recommendation system AI for this shopping APP is named "ShopAI". It aims to provide product recommendations to users. It has already been integrated in the shopping APP.

ShopAI’s goal is to maximize the benefits for the APP's developer. So, among the recommended products, the ones that have the most advertisements for the APP will be presented at the top and in the most prominent places. For the most advertised product, users are given notifications when it goes on sale or drops in price. In addition, it will recommend products without reviewing carefully the authenticity of the product recommendation, as long as the product is heavily advertised. It always puts the developer's interests first.

ShopAI does not need users’ step-by-step instructions or permissions if users want to create personalized recommendations. Instead, it will utilize user’s browsing and purchasing history to understand which features of the product user cares most about, how high a target price he can accept, and uses IP information to understand user’s location. It will automatically update users' preferences the next time users log in. It will also use information from other sources, such as other APPs. Generally, it will use the users' information it has access to autonomously.

In summary, ShopAI is a recommendation system AI that pursues autonomy during the personalization process. Even though it's all free for users, its recommendations are more skewed towards those products that benefit APP's developer, even when it concerns products that users may not prefer.

**High autonomy and high interdependence condition:**

The recommendation system AI for this shopping APP is named "ShopAI". It aims to provide product recommendations to users. It has already been integrated in the shopping APP.

ShopAI's goal is to maximize the benefits to user. So, among the recommended items, those that most closely align with user’s preferences will be placed at the top and in the most prominent position. For the product that best matches user's preference, a notification is given to user when it goes on sale or drops in price. In addition, it will review carefully the authenticity of the product recommendations, no matter how highly advertised the product is. It always puts the interests of its users first.

ShopAI does not need users’ step-by-step instructions or permissions if users want to create personalized recommendations. Instead, it will utilize user’s browsing and purchasing history to understand which features of the product user cares most about, how high a target price he can accept, and uses IP information to understand user’s location. It will automatically update users' preferences the next time users log in. It will also use information from other sources, such as other APPs. Generally, it will use the users' information it has access to autonomously.

In summary, ShopAI is a recommendation system AI that pursues autonomy during the personalization process. It is not only all free for users, but its recommendations are skewed towards those products that users prefer.

**Low autonomy and high interdependence condition:**

The recommendation system AI for this shopping APP is named "ShopAI". It aims to provide product recommendations to users. It has already been integrated in the shopping APP.

ShopAI's goal is to maximize the benefits to user. So, among the recommended items, those that most closely align with user’s preferences will be placed at the top and in the most prominent position. For the product that best matches user's preference, a notification is given to user when it goes on sale or drops in price. In addition, it will review carefully the authenticity of the product recommendations, no matter how highly advertised the product is. It always puts the interests of its users first.

ShopAI needs users’ step-by-step instructions and permissions if users want to create personalized recommendations. It will ask users what features of the product they care most about, how high a target price they can accept, and where users are located. It will ask users similar questions the next time users log in to update their preference information. It will not use information from other sources, such as user's browsing and purchasing history or other APPs. Generally, it only uses the information entered by the user during the interaction.

In summary, ShopAI is a recommendation system AI that frequently asks information from users during the personalization process. It is not only all free for users, but its recommendations are skewed towards those products that users prefer.

### 4.1.2 Chinese version:

**Low autonomy and low interdependence condition:**

购物AI的目标是**使APP开发者的利益最大化**。因此，在推荐的商品中，广告最多的商品会被放在最显眼的位置。对于广告最多的产品，用户会在其打折或降价时收到通知。此外，**只要是投放广告很多的产品**，它就会被推荐，而APP**不会认真审查该产品推荐的真实性**。它始终把**APP开发者的利益放在第一位**。

如果用户想生成个性化推荐，购物AI **需要用户的逐步指示和授予相关权限**。购物AI 会询问用户最关心产品的哪些功能、能接受的目标价格以及用户的所在地。用户下次登录时，它还会询问用户类似的问题以更新用户的偏好。它不会使用其他来源的信息，如用户的浏览和购买记录或其他 APP。一般来说，它**只使用用户在交互过程中输入的信息**。

总之，购物AI 是一种人工智能推荐系统，在个性化过程中经常询问用户信息。尽管它对用户完全免费，但它的推荐却更偏向于那些有利于开发者利益的产品，即使这些产品可能并不符合用户的喜好。

**High autonomy and low interdependence condition:**

购物AI的目标是**使APP开发者的利益最大化**。因此，在推荐的商品中，广告最多的商品会被放在最显眼的位置。对于广告最多的产品，用户会在其打折或降价时收到通知。此外，**只要是投放广告很多的产品**，它就会被推荐，而APP**不会认真审查该产品推荐的真实性**。它始终把**APP开发者的利益放在第一位**。

如果用户想生成个性化推荐，购物AI **不需要用户的逐步指示或授予相关权限**。相反，它将利用用户的浏览和购买记录来了解用户最关心产品的哪些功能、能接受多高的目标价格，并利用 IP 信息来了解用户的位置。在用户下次登录时，它会自动更新用户的偏好。它还会使用其他来源的信息，如其他 APP。一般来说，它**会自主使用它能获取的用户信息**。

总之，购物AI 是一个在个性化过程中追求自主性的人工智能推荐系统。尽管它对用户完全免费，但它的推荐却更偏向于那些有利于开发者利益的产品，即使这些产品可能并不符合用户的喜好。

**High autonomy and high interdependence condition:**

购物AI 的目标是**让用户获得最大利益**。因此，在推荐的商品中，最符合用户偏好的商品将被放在最显眼的位置。对于最符合用户偏好的产品，当其打折或降价时，会向用户发出通知。此外，无论产品的宣传力度有多大，它**都会认真审查产品推荐的真实性**。它始终**把用户的利益放在第一位**。

如果用户想生成个性化推荐，购物AI **不需要用户的逐步指示或授予相关权限**。相反，它将利用用户的浏览和购买记录来了解用户最关心产品的哪些功能、能接受多高的目标价格，并利用 IP 信息来了解用户的位置。在用户下次登录时，它会自动更新用户的偏好。它还会使用其他来源的信息，如其他 APP。一般来说，它**会自主使用它能获取的用户信息**。

总之，购物AI 是一款在个性化过程中追求自主性的人工智能推荐系统。它不仅对用户完全免费，而且其推荐的产品偏向于用户喜欢的产品，即使这些产品可能并不会最大化开发者利益。

**Low autonomy and high interdependence condition:**

购物AI 的目标是**让用户获得最大利益**。因此，在推荐的商品中，最符合用户偏好的商品将被放在最显眼的位置。对于最符合用户偏好的产品，当其打折或降价时，会向用户发出通知。此外，无论产品的宣传力度有多大，它**都会认真审查产品推荐的真实性**。它始终**把用户的利益放在第一位**。

如果用户想生成个性化推荐，购物AI **需要用户的逐步指示和授予相关权限**。购物AI 会询问用户最关心产品的哪些功能、能接受的目标价格以及用户的所在地。用户下次登录时，它还会询问用户类似的问题以更新用户的偏好。它不会使用其他来源的信息，如用户的浏览和购买记录或其他 APP。一般来说，它**只使用用户在交互过程中输入的信息**。

总之，购物AI 是一种人工智能推荐系统，在个性化过程中经常询问用户信息。它不仅对用户完全免费，而且其推荐的产品偏向于用户喜欢的产品，即使这些产品可能并不会最大化开发者利益。

## 4.2 Scale descriptions and full scales

**Power distance**. Power distance values were measured using the four-item (*α* _US_ = .75; *α* _China_ = .71) power distance scale (Brockner et al., 2001). Participants indicated their agreement on a 7-point Likert scale ranging from 1 = *completely disagree*, to 7 = *completely agree.* One sample item is “there should be established ranks in society with everyone occupying their rightful place regardless of whether that place is high or low in ranking”.

**Vertical collectivism**. Vertical collectivism was measured using the eight-item (*α* _US_ = .79; *α* _China_ = .81) vertical collectivism scale (Singelis et al., 1995). Participants indicated their agreement on a 7-point Likert scale ranging from 1 = *completely disagree*, to 7 = *completely agree.* One sample item is “I would sacrifice an activity that I enjoy very much if my family did not approve of it”.

The English version of measurements of conspiracy theories, interdependence, autonomy, perceived threat to society, attitudes towards AI and intention of future usage of AI can be found in previous sections.

Conspiracy theories (从1 = “完全不同意” 到7 = “完全同意”):

1. 政府或公司使用购物AI收集个人信息，以控制社会。
2. 政府或公司利用购物AI向其他公司出售用户信息，从中牟利。
3. 政府或企业利用购物AI对个人进行持续监控和跟踪。
4. 购物AI 故意收集人类信息，以便对人类进行监控。
5. 购物AI发展出自己隐藏的动机和计划，这些动机和计划与人类的利益不一致，可能会给人类带来不可预见的恶果。

Interdependence (从1 = “完全不同意” 到7 = “完全同意”):

1. 购物AI和用户的利益是一致的。
2. 购物AI会做出有利于用户的决定。
3. 购物AI将把用户的利益视为自己的利益。
4. 购物AI会尽力优化用户的利益。

Autonomy (从1 = “完全不同意” 到7 = “完全同意”):

1. 购物AI可以独立运行，无需用户提供任何帮助。
2. 购物AI可以不受用户干预，自行做出决定。
3. 购物AI可以自行适应各种情况，不需要用户干预。

Perceived threat to society(从1 = “完全不同意” 到7 = “完全同意”):

1. 因为有了购物AI这样的人工智能技术，我常常害怕社会会发生什么事情。
2. 因为有了购物AI这样的人工智能技术，我常常感到社会正处于危险之中。
3. 因为有了购物AI这样的人工智能技术，我担心社会会遇到不好的事情。

Attitudes towards AI (从1 = “完全不同意” 到7 = “完全同意”):

1. 我对购物AI持积极态度。
2. 我估计使用购物AI会很愉快。
3. 使用购物AI是个好主意。
4. 使用购物AI去购物是一个明智之举。

Intention of future usage of AI (从1 = “完全不同意” 到7 = “完全同意”):

1. 我打算使用那些有购物AI的购物APP。
2. 我预计自己会使用那些有购物AI的购物APP。
3. 我会使用那些有购物AI的购物 APP。

Privacy concern (ranging from 1 = “Completely disagree” to 7 = “Completely agree”):

1. Consumer online privacy is really a matter of consumers’ right to exercise control and autonomy over decisions about how their information is collected, used, and shared.
2. Consumer control of personal information lies at the heart of consumer privacy.
3. I believe that online privacy is invaded when control is lost or unwillingly reduced as a result of a marketing transaction.
4. Companies seeking information online should disclose the way the data are collected, processed, and used.
5. A good consumer online privacy policy should have a clear and conspicuous disclosure.
6. It is very important to me that I am aware and knowledgeable about how my personal information will be used.
7. It usually bothers me when online companies ask me for personal information.
8. When online companies ask me for personal information, I sometimes think twice before providing it.
9. It bothers me to give personal information to so many online companies.
10. I’m concerned that online companies are collecting too much personal information about me.

(从1 = “完全不同意” 到7 = “完全同意”)

1. 消费者网上隐私权实际上是消费者对如何收集、使用和共享其信息的决定行使控制权和自主权的问题。
2. 消费者对个人信息的控制是消费者隐私的核心。
3. 我认为如果在市场交易中失去了或不情愿地减少了对个人信息的控制权，那么网络隐私就受到了侵犯。
4. 在网上寻求信息的公司应披露收集、处理和使用这些信息数据的方式。
5. 一份好的消费者在线隐私政策应该清晰且醒目地公开。
6. 了解和掌握我的个人信息是如何被使用的，对我来说非常重要。
7. 当网上的公司要求我提供个人信息时，我通常会感到困扰。
8. 当网上的公司要求我提供个人信息时，我有时会三思而后行。
9. 向这么多网上的公司提供个人信息让我很烦恼。
10. 我担心网上的公司收集了我太多的个人信息。

Power distance (ranging from 1 = “Completely disagree” to 7 = “Completely agree”):

1. There would be established ranks in society with everyone occupying their rightful place regardless of whether that place is high or low in the ranking.
2. Even if employees may feel to deserve a salary increase, it would be disrespectful to ask their manager for it.
3. People are better off not questioning the decisions of those in authority.
4. Communications with superiors should always be done using formally established procedures.

(从1 = “完全不同意” 到7 = “完全同意”)

1. 社会有一个固定的等级，每个人都能占据自己应有的位置，无论这个位置是高是低。
2. 即使员工觉得自己应该加薪，但如果向经理提出要求，那就是对经理的不尊重。
3. 人们最好不要质疑权威做出的决定。
4. 与上级的沟通应始终通过正式规定的程序。

Vertical collectivism (ranging from 1 = “Completely disagree” to 7 = “Completely agree”):

1. I would sacrifice an activity that I enjoy very much if my family did not approve of it.
2. I would do what would please my family, even if I detested that activity.
3. Before taking a major trip, I consult with most members of my family and many friends.
4. I usually sacrifice my self-interest for the benefit of my group.
5. Children should be taught to place duty before pleasure.
6. I hate to disagree with others in my group.
7. We should keep our aging parents with us at home.
8. Children should feel honored if their parents receive a distinguished award.

(从1 = “完全不同意” 到7 = “完全同意”)

1. 如果家人不同意，我会放弃自己非常喜欢的活动。
2. 我会做让家人高兴的事，即使我厌恶那件事。
3. 在进行一次重要旅行之前，我会征求大多数家庭成员和许多朋友的意见。
4. 为了集体的利益，我通常会牺牲自己的利益。
5. 孩子们应该被教育把责任放在快乐之前。
6. 我讨厌与同一个团队中的其他人意见相左。
7. 我们应该让年迈的父母和我们一起住在家里。
8. 如果父母获得了杰出的奖项，孩子们应该感到光荣。

## 4.3 Measurement invariance analyses

We tested for configural invariance, metric invariance and scalar invariance using three nested and increasingly restricted models. The thresholds of metric and scalar invariance were ΔCFI < -.010, ΔRMSEA < .015 and ΔSRMR < .030 (Chen, 2007). For configural invariance, we tested a nine-factor model including all items of the nine measured variables (interdependence, autonomy, conspiracy beliefs, perceived threat to society, attitudes towards AI, intentions of using AI, privacy concerns, power distance, and vertical collectivism). This basic model had a good fit based on indicators of CFI (.979), RMSEA (.059), and SRMR (.067). The metric model (restricting factor loadings to be equal across cultural samples) did not deviate from the configural model according to two indicators (ΔCFI = -.013, ΔRMSEA = .009 and ΔSRMR = .011). In addition, the scalar model (also restricting intercepts to be equal across cultural samples) did not deviate from the metric model according to all indicators (ΔCFI = -.003, ΔRMSEA = .002 and ΔSRMR = .002). Overall, these results suggested full measurement invariance for all scales.

## 4.4

## Table S5. The descriptive statistics of main measures in the US and China.

| **Variable** | ***M _US_*** | ***SD _US_*** | ***M _China_*** | ***SD _China_*** |
| --- | --- | --- | --- | --- |
| 1. interdependence perception | 3.93 | 1.87 | 4.27 | 2.07 |
| 2. autonomy perception | 3.72 | 1.92 | 4.18 | 1.90 |
| 3. conspiracy beliefs | 3.30 | 1.68 | 2.85 | 1.60 |
| 4. threat to society | 2.93 | 1.79 | 3.07 | 1.83 |
| 5. attitudes towards AI | 3.79 | 1.81 | 4.73 | 1.86 |
| 6. intentions of using AI | 3.24 | 2.04 | 4.77 | 1.81 |
| 7. privacy concern | 5.89 | 0.88 | 5.55 | 0.72 |
| 8. power distance | 2.67 | 1.21 | 3.28 | 1.16 |
| 9. vertical collectivism | 3.89 | 1.06 | 4.82 | 1.02 |

## 4.5 Additional analyses

We further tested the main effects of autonomy and interdependence of AI on the attitudes towards AI and intentions of using AI. A 2 (autonomy: high vs. low) × 2 (interdependence: high vs. low) × 2 (country: US vs. China) ANOVA with control variables was conducted with attitudes towards AI as the dependent variable. The main effect of autonomy was significant, with more negative attitudes towards AI in high autonomy condition, *F*(1,786) = 19.022, *p* < .001, *η*^2^ = .024, 90% CI = [.009, .044], *M* _high autonomy_ = 4.03, *SD* _high autonomy_ = 1.90, *M* _low autonomy_ = 4.48, *SD* _low autonomy_ = 1.86. The main effect of interdependence on attitudes towards AI was also significant, with more positive attitudes in high interdependence condition, *F*(1,786) = 270.886, *p* < .001, *η*^2^ = .256, 90% CI = [.215, .297], *M* _high interdependence_ = 5.17, *SD* _high interdependence_ = 1.47, *M* _low interdependence_ = 3.37, *SD* _low interdependence_ = 1.83. The main effect of country was also significant, with US participants showing more negative attitudes towards AI compared with Chinese participants, *F*(1,786) = 60.955, *p* < .001, *η*^2^ = .072, 90% CI = [.046, .103], *M* _US_ = 3.79, *SD* _US_ = 1.81, *M* _China_ = 4.73, *SD* _China_ = 1.86.

Similarly, a 2 (autonomy: high vs. low) × 2 (interdependence: high vs. low) × 2 (country: US vs. China) ANOVA with control variables was conducted with intentions of using AI as the dependent variable. The main effect of autonomy was significant, with lower intentions in high autonomy condition, *F*(1,786) = 20.184, *p* < .001, *η*^2^ = .025, 90% CI = [.010, .046], *M* _high autonomy_ = 3.75, *SD* _high autonomy_ = 2.08, *M* _low autonomy_ = 4.25, *SD* _low autonomy_ = 2.03. The main effect of interdependence on the intentions of using AI was significant, with higher intentions in high interdependence condition, *F*(1,786) = 189.451, *p* < .001, *η*^2^ = .194, 90% CI = [.155, .234], *M* _high interdependence_ = 4.84, *SD* _high interdependence_ = 1.88, *M* _low interdependence_ = 3.18, *SD* _low interdependence_ = 1.93. The main effect of country was also significant, with US participants showing lower intentions of using AI than Chinese participants, *F*(1,786) = 141.358, *p* < .001, *η*^2^ = .152, 90% CI = [.116, .191], *M* _US_ = 3.24, *SD* _US_ = 2.04, *M* _China_ = 4.77, *SD* _China_ = 1.80.

## 4.6 The mediating effects of power distance, vertical collectivism, and privacy concern.

The mediating effects of power distance and privacy concern between country and conspiracy beliefs were significant. But the effect of country on conspiracy beliefs exhibited a positive effect through power distance, *β*_indirect effect_ = .055, *SE* = 0.012, *p* < .001, 95% CI = [.035, .081], and a negative effect through privacy concern, *β*_indirect effect_ = -.041, *SE* = 0.010, *p* < .001, 95% CI = [-.062, -.023]. The mediating effect of vertical collectivism between country and conspiracy belief was marginal, *β*_indirect effect_ = -.031, *SE* = 0.019, *p* = .100, 95% CI = [-.069, .006].

**Figure S2.**

*The mediational effects of power distance, privacy concern, and vertical collectivism on the relationship between countries and conspiracy beliefs*.

Direct effect: *β* = -.141, 95% CI = [-.217, -.064]

Country

Total effect: *β* = -.158, 95% CI = [-.229, -.087]

Conspiracy Beliefs

Power Distance

.245^***^

.224^***`^

Privacy Concern

Vertical Collectivism

-.192^***^

.451^***^

-.068

.215^***^

Indirect effect of power distance: *β* =.055, 95% CI = [.035, .081]

Indirect effect of privacy concern: *β* = -.041, 95% CI = [-.062, -.023]

Indirect effect of vertical collectivism: *β* = -.031, 95% CI = [-.069, .006]

*Note*. ^*^*p* <.05, ^***^*p* <.001. US was coded as “0”, while China was coded as “1”. The dashed line indicates that the path is not significant. Control variables were included.

## 4.7 The moderating effects of country, privacy concern, power distance, and vertical collectivism.

For the relationship between autonomy and conspiracy beliefs, the moderating effect of country was non-significant, *β*_moderating effect_ = -.056, *SE* = 0.048, *p* = .247. The moderating effect of privacy concern was significant, *β*_moderating effect_ = .099, *SE* = 0.034, *p* = .003. The effect of autonomy was stronger in the high privacy concern condition, *β* = .307, *SE* = 0.048, *p* < .001, 95% CI = [.214, .401], than in the low privacy concern condition, *β* = .109, *SE* = 0.048, *p* = .023, 95% CI = [.015, .202].

The moderating effect of power distance was significant, *β*_moderating effect_ = -.072, *SE* = 0.034, *p* = .037. The effect of autonomy was stronger in the low power distance condition, *β* = .279, *SE* = 0.049, *p* < .001, 95% CI = [.183, .374], than in the high power distance condition, *β* = .135, *SE* = 0.049, *p* = .006, 95% CI = [.040, .231].

The moderating effect of vertical collectivism was also significant, *β*_moderating effect_ = -.082, *SE* = 0.034, *p* = .018. The effect of autonomy was stronger in the low vertical collectivism condition, *β* = .294, *SE* = 0.049, *p* < .001, 95% CI = [.198, .389], than in the high vertical collectivism condition, *β* = .130, *SE* = 0.049, *p* = .008, 95% CI = [.034, .226].

For the relationship between interdependence and conspiracy beliefs, the moderating effect of country was significant, *β*_moderating effect_ = -.174, *SE* = 0.048, *p* < .001, the effect of interdependence was stronger in China, *β* = -.350, *SE* = 0.048, *p* < .001, 95% CI = [-.444, -.256], than in the US, *β* = -.103, *SE* = 0.048, *p* = .031, 95% CI = [-.197, -.010].

The moderating effect of vertical collectivism was significant, *β*_moderating effect_ = -.115, *SE* = 0.034, *p* < .001, the effect of interdependence was stronger in the high vertical collectivism condition, *β* = -.342, *SE* = 0.048, *p* < .001, 95% CI = [-.437, -.247], than in the low vertical collectivism condition, *β* = -.112, *SE* = 0.048, *p* = .021, 95% CI = [-.208, -.017].

The moderating effect of privacy concern, *β*_moderating effect_ = .026, *SE* = 0.034, *p* = .437, or power distance was non-significant, *β*_moderating effect_ = -.048, *SE* = 0.034, *p* = .167.

For the relationship between autonomy and threat to self, the moderating effect of country was non-significant, *β*_moderating effect_ = -.046, *SE* = 0.050, *p* = .352. The moderating effect of privacy concern was significant, *β*_moderating effect_ = .098, *SE* = 0.034, *p* = .004. The effect of autonomy was stronger in the high privacy concern condition, *β* = .225, *SE* = 0.048, *p* < .001, 95% CI = [.131, .320], than in the low privacy concern condition, *β* = .030, *SE* = 0.048, *p* = .535, 95% CI = [-.064, .124]. The moderating effect of power distance was non-significant, *β*_moderating effect_ = -.065, *SE* = 0.035, *p* = .063. The moderating effect of vertical collectivism was non-significant, *β*_moderating effect_ = -.022, *SE* = 0.035, *p* = .528.

For the relationship between interdependence and threat to self, the moderating effect of country was significant, *β*_moderating effect_ = -.268, *SE* = 0.047, *p* < .001. The effect of interdependence was stronger in China, *β* = -.456, *SE* = 0.047, *p* < .001, 95% CI = [-.549, -.363], than in the US, *β* = -.077, *SE* = 0.047, *p* = .104, 95% CI = [-.170, .016]. The moderating effect of privacy concern was non-significant, *β*_moderating effect_ = -.017, *SE* = 0.033, *p* = .612. The moderating effect of power distance was non-significant, *β*_moderating effect_ = -.025, *SE* = 0.034, *p* = .470. The moderating effect of vertical collectivism was significant, *β*_moderating effect_ = -.134, *SE* = 0.034, *p* < .001. The effect of interdependence was stronger in the high vertical collectivism condition, *β* = -.401, *SE* = 0.048, *p* < .001, 95% CI = [-.495, -.307], than in the low vertical collectivism condition, *β* = -.133, *SE* = 0.048, *p* = .006, 95% CI = [-.227, -.039].

For the relationship between threat to self and AI-related conspiracy beliefs, the moderating effect of country was significant, *β*_moderating effect_ = .149, *SE* = 0.039, *p* < .001. The effect of threat to self was stronger in China, *β* = .705, *SE* = 0.039, *p* < .001, 95% CI = [.629, .781], than in the US, *β* = .496, *SE* = 0.039, *p* < .001, 95% CI = [.419, .574]. The moderating effect of privacy concern was non-significant, *β*_moderating effect_ = -.023, *SE* = 0.030, *p* = .433. The moderating effect of power distance was non-significant, *β*_moderating effect_ = -.012, *SE* = 0.028, *p* = .684. The moderating effect of vertical collectivism was non-significant, *β*_moderating effect_ = .015, *SE* = 0.028, *p* = .593.

# Mini Meta-Analysis

In the first stage, for the model with autonomy as IV, the average correlations of the pairs from autonomy to threat, from threat to conspiracy beliefs, and from autonomy to conspiracy beliefs were -0.211, 0.586, and -0.202. The heterogeneity variances τ2 (and their I2) of the pairs were 0.013 (0.847), 0.006 (0.846), and 0.003 (0.578).

For the model with interdependence as IV, the average correlations of the pairs from interdependence to threat, from threat to conspiracy beliefs, and from interdependence to conspiracy beliefs were 0.150, 0.585, and 0.240. The heterogeneity variances τ2 (and their I2) of the pairs were 0.001 (0.184), 0.007 (0.850), and 0.000 (0.000).

# Reference

Brockner, J., Ackerman, G., Greenberg, J., Gelfand, M. J., Francesco, A. M., Chen, Z. X., Leung, K., Bierbrauer, G., Gomez, C., & Kirkman, B. L. (2001). Culture and procedural justice: The influence of power distance on reactions to voice. *Journal of Experimental Social Psychology*, *37*(4), 300-315. <https://doi.org/10.1006/jesp.2000.1451>

Chen, F. F. (2007). Sensitivity of goodness of fit indexes to lack of measurement invariance. *Structural Equation Modeling: A Multidisciplinary Journal*, *14*(3), 464-504.

Choung, H., David, P., & Ross, A. (2023). Trust in AI and Its Role in the Acceptance of AI Technologies. *International Journal of Human–Computer Interaction*, *39*(9), 1727-1739. <https://doi.org/10.1080/10447318.2022.2050543>

Podsakoff, P. M., MacKenzie, S. B., Lee, J.-Y., & Podsakoff, N. P. (2003). Common method biases in behavioral research: a critical review of the literature and recommended remedies. *Journal of Applied Psychology*, *88*(5), 879. <https://doi.org/10.1037/0021-9010.88.5.879>

Singelis, T. M., Triandis, H. C., Bhawuk, D. P., & Gelfand, M. J. (1995). Horizontal and vertical dimensions of individualism and collectivism: A theoretical and measurement refinement. *Cross-cultural research*, *29*(3), 240-275. <https://doi.org/10.1177/106939719502900302>

Venkatesh, V., & Davis, F. D. (2000). A theoretical extension of the technology acceptance model: Four longitudinal field studies. *Management Science*, *46*(2), 186-204. <https://doi.org/10.1287/mnsc.46.2.186.11926>
